# The paradox of neglecting changes in behavior: How standard epidemic models misestimate both transmissibility and final epidemic size

**DOI:** 10.1371/journal.pcbi.1014746

**Published:** 2026-09-03

**Authors:** Binod Pant, Marko Lalovic, István Z. Kiss, Mauricio Santillana

**Affiliations:** 1 Department of Mathematics and Statistics, Mississippi State University, Jackson, Mississippi, United States of America; 2 Machine Intelligence Group for the Betterment of Health and the Environment, Northeastern University, Boston, Massachusetts, United States of America; 3 Network Science Institute, Northeastern University, Boston, Massachusetts, United States of America; 4 Network Science Institute, Northeastern University London, London, United Kingdom; 5 Department of Mathematics, Northeastern University, Boston, Massachusetts, United States of America; 6 Department of Epidemiology, Harvard T.H. Chan School of Public Health, Boston, Massachusetts, United States of America; Queen Mary University of London, UNITED KINGDOM OF GREAT BRITAIN AND NORTHERN IRELAND

## Abstract

During epidemic outbreaks, populations adapt their behavior in response to disease burden, fundamentally altering transmission dynamics. Despite this, most compartmental models assume constant contact rates throughout outbreaks. To quantify biases from this assumption, we fitted a baseline SEIRD model with constant transmission and three behavioral variants—incorporating mortality-driven transmission reduction via exponential, rational, and mixed functional forms—to COVID-19 mortality data from 20 selected US locations during the first pandemic wave (March–July 2020). All three behavioral models achieved a lower median normalized sum of squared error in at least 18 of 20 locations, and Bayesian model selection favored them in at least 18 of 20 locations. More importantly, we identified systematic biases when behavioral responses are ignored: the baseline model consistently underestimated the basic reproduction number (${ℛ}_{0}$) while paradoxically overestimating the final epidemic size. Median ${ℛ}_{0}$ estimates from the behavioral models exceeded the baseline estimates across all 20 locations, yet baseline models predicted larger cumulative infection burdens. Controlled synthetic experiments—where mortality trajectories were generated from behavioral models with known parameters—confirmed these biases result from model misspecification rather than data quality or stochastic variation. We prove analytically that for any fixed ${ℛ}_{0}$, the baseline model overestimates cumulative infections compared to behavioral models where mortality reduces transmission, regardless of functional form. This dual bias has potential implications for pandemic response: standard models may simultaneously underestimate pathogen contagiousness, which could contribute to delayed or insufficient early interventions while overestimating infection burden, which could bias planning for later epidemic phases. Our findings across 20 geographically diverse locations demonstrate that incorporating behavioral change substantially improves both model fit and estimation of epidemiological parameters relevant for public health policy.

## Author summary

When diseases spread, people change their behavior—avoiding crowds, wearing masks, washing hands more frequently. Yet many mathematical models used to predict epidemics assume people behave the same way throughout an outbreak. We asked: What happens when models ignore these behavioral changes? Using COVID-19 data from 20 selected US locations, we compared a traditional model, which assumes constant transmission, against models where transmission decreases as deaths increase. We found a troubling paradox: models ignoring behavior consistently suggest diseases are less contagious than estimates from behavioral models, yet simultaneously predict that more people will ultimately become infected. This mismatch matters for decision-making: underestimating how easily a disease spreads could contribute to delayed or insufficient early interventions, while overestimating total infections could bias planning for later epidemic phases. We confirmed this paradox through computer simulations where we knew the true answer, and showed analytically that behavioral adaptation reduces final epidemic size when the basic reproduction number is held constant. Our work shows that incorporating human behavioral responses is not just a modeling refinement—it is important for epidemic predictions that inform public health decisions.

## 1. Introduction

Behavioral responses to epidemic threats fundamentally shape disease transmission. During the 1918 influenza pandemic, communities closed schools, banned public gatherings, and adopted mask-wearing to reduce transmission [1,2]. The HIV/AIDS epidemic saw widespread adoption of condom use, averting an estimated 100 million infections between 1990 and 2019 [3]. More recently, the COVID-19 pandemic triggered rapid behavioral shifts, including masking and social distancing, with mask mandates alone associated with significant reductions in cases and deaths [4,5]. Recent survey data from the United States suggests that human behavior, which varied spatially and temporally during COVID-19, was strongly associated with changes in COVID-19 mortality and hospitalization [6], indicating that observed disease severity indicators drive behavioral adaptation. These examples demonstrate that human populations do not passively experience epidemics—they adapt their behavior in response to perceived risk, fundamentally altering transmission dynamics.

Compartmental models based on Susceptible-Exposed-Infected-Recovered (SEIR) frameworks have been widely used to understand epidemic trajectories and guide public health interventions [7]. Despite historical and contemporary evidence showing that people actively adapt their behavior in response to disease threats, most models assume populations passively experience outbreaks by maintaining constant contact patterns. Although the assumption of constant transmission rates is mathematically convenient, it ignores the dynamic feedback between disease burden and behavioral change. Recent modeling efforts have begun incorporating behavioral responses through various mechanisms, including prevalence-dependent [8–11], hospitalization-driven [12], and mortality-driven feedback [8,13–15]. LeJeune et al. [16] classify these approaches as either endogenous (behavior changes as a function of disease location variables within the model, creating a feedback loop) or exogenous (behavior changes through fixed or time-varying parameters informed by external data sources such as government policies and mobility patterns, or through modelers’ assumptions, rather than a location variable within a model itself).

The critical question is whether systematic biases arise when models fail to account for behavioral change. If populations adapt their behavior in response to disease burden—as historical and contemporary evidence suggests—then models assuming constant transmission may systematically mischaracterize epidemic dynamics. Such mischaracterization could have serious consequences for public health decision-making: underestimating pathogen transmissibility might lead to insufficient interventions, while overestimating infection burden could result in misallocated resources. Despite these stakes, few studies have systematically investigated—either numerically or analytically—whether and to what extent omitting behavioral feedback introduces biases in epidemic models. Recent work has demonstrated that incorporating behavioral responses improves model fit to observed data [8,12,14,17,18], with behavioral models capturing epidemic trajectories better than their behavior-free counterparts. Behavioral models have also shown superior performance in out-of-sample validation [8,12]. Theoretical studies have examined how behavioral mechanisms—including vaccination behavior [19], awareness-driven protection [20], and incidence-dependent contact reduction [9]—alter epidemic outcomes such as cumulative infection burden; notably, models ignoring behavior change have systematically overshot final size projections [21]. Furthermore, using a 10-dimensional compartmental model fitted to a single US location, Pant et al. [8] show that models ignoring behavioral change underestimate the basic reproduction number relative to models that explicitly incorporate it; whether this finding generalizes across locations and simpler model structures motivates the present study. However, systematic comparison of key epidemiological quantities—such as basic reproduction number and final size—estimated through models with versus without behavioral feedback, using empirical data across many locations, remains lacking.

We address this gap by systematically comparing compartmental models with and without behavioral feedback, fitted to COVID-19 mortality data from 20 US locations (19 U.S. states and the District of Columbia) during the first pandemic wave (March–July 2020). We implement three behavioral models where the transmission rate decreases as mortality increases, alongside a baseline model with constant transmission. Using Bayesian methods, we fit each model to observed mortality trajectories, estimate posterior distributions for the basic reproduction number and final epidemic size, and compare model performance both qualitatively and quantitatively. To test whether biases arise from model misspecification rather than data limitations, we conduct controlled synthetic experiments where true parameter values are known by design. We also analytically derive the relationship between final epidemic size in baseline versus behavioral models when the basic reproduction number is identical.

Our analysis reveals three key findings with important public health implications. First, behavioral models consistently outperform baseline models across nearly all locations examined, as evidenced by both qualitative and quantitative comparisons — a finding consistent with existing works in the literature [8,12,14,18]. Second, baseline models systematically underestimate the basic reproduction number while counterintuitively overestimating the total proportion of the population infected compared to all three behavioral model formulations. We confirm this dual bias through synthetic experiments, demonstrating that these biases emerge when behavioral feedback is present in the data but models do not explicitly account for behavioral change — a finding that, to our knowledge, has not been previously demonstrated by fitting to empirical data across multiple locations or confirmed through controlled synthetic experiments. Third, for the class of models considered in this study, we prove analytically that for a fixed basic reproduction number, models that ignore behavior change overestimate the final epidemic size in comparison to model that explicitly accounts for behavior change — extending prior theoretical results for specific SIR formulations [21]. Overall, these findings demonstrate that incorporating behavioral dynamics is not merely a modeling refinement but essential for accurate epidemic parameter estimation and evidence-based pandemic response.

This paper is organized as follows. In Section 2, we formulate the baseline model that does not explicitly account for behavioral change and three behavioral model variants, describe the COVID-19 mortality data used for calibration, and outline our Bayesian parameter inference approach. Section 3 presents our three key findings: behavioral models’ superior fit to observed data, systematic underestimation of the basic reproduction number by baseline models, and overestimation of infection burden when behavioral responses are ignored. We support these findings through both real data analysis across 20 US locations and controlled synthetic experiments. Section 4 discusses the public health implications and limitations of our work.

## 2. Methods

### 2.1 Model formulation

We developed a compartmental SEIRD model incorporating behavioral responses to disease-induced mortality. The population is stratified into susceptible (*S*), exposed (*E*), infectious (*I*), recovered (*R*), and deceased (*D*) compartments, with total living population *N*(*t*) = *S*(*t*) + *E*(*t*) + *I*(*t*) + *R*(*t*) at time *t*. The model dynamics are governed by the following system of differential equations:


$$\begin{array}{cc} \frac{dS}{dt} & =-\beta (t)\frac{SI}{N},\\ \frac{dE}{dt} & =\beta (t)\frac{SI}{N}-\sigma E,\\ \frac{dI}{dt} & =\sigma E-(\gamma +\delta )I,\\ \frac{dR}{dt} & =\gamma I,\\ \frac{dD}{dt} & =\delta I, \end{array}$$ (2.1)

where σ is the incubation rate, γ is the recovery rate, and δ is the disease-induced mortality rate. Under homogeneous mixing, the transmission rate β(t)=c(t)·p, where *c*(*t*) is the per-capita contact rate and *p* (assumed to be constant) is the probability of transmission per contact. Behavioral adaptation *t*hat reduces contacts thus enters the model directly as a reduction in β(t). The transmission rate β(t) varies between model variants to capture different behavioral assumptions, whereby the severity of the outbreak is assumed to lead to behavioral adaptation aimed at reducing infection risk.

#### Baseline model.

The baseline model assumes constant transmission $\beta (t)=\beta_{0}$, representing traditional SEIRD dynamics without explicit behavioral feedback.

#### Behavioral models.

Behavioral models incorporate dynamic feedback through mortality-dependent transmission, with β(t) declining as deaths increase to reflect behavioral adaptation to perceived risk. The transmission rate β(t) depends on three quantities: $\beta_{0}$, the baseline transmission rate in the absence of behavioral response; δI(t), the instantaneous death rate due to disease; and ζ, which quantifies the population’s sensitivity to mortality. Larger values of ζ correspond to stronger behavioral responses. We consider three functional forms of β(t):

(a) *Exponential form:*

$$\begin{array}{c} \beta (t)=\beta_{0}\;e^{-\zeta \;\delta I(t)}, \end{array}$$ (2.2)

where transmission rate decays exponentially with increasing mortality.

(b) *Rational form:*

$$\begin{array}{c} \beta (t)=\frac{\beta_{0}}{1+\zeta \;\delta I(t)}, \end{array}$$ (2.3)

which produces similar behavior to the exponential form but saturates more slowly as deaths rise.

(c) *Mixed form:*

$$\begin{array}{c} \beta (t)=\frac{\beta_{0}e^{-\zeta \;\delta I(t)}}{1+\zeta \;\delta I(t)}, \end{array}$$ (2.4)

which incorporates both exponential and rational forms. This hybrid form saturates faster than either individual form as mortality increases.

All behavioral models reduce to the baseline model when ζ=0. Hereafter, we refer to these as the *Behavioral (Exponential)*, *Behavioral (Rational)*, and *Behavioral (Mixed)* models.

**Fig 1:**
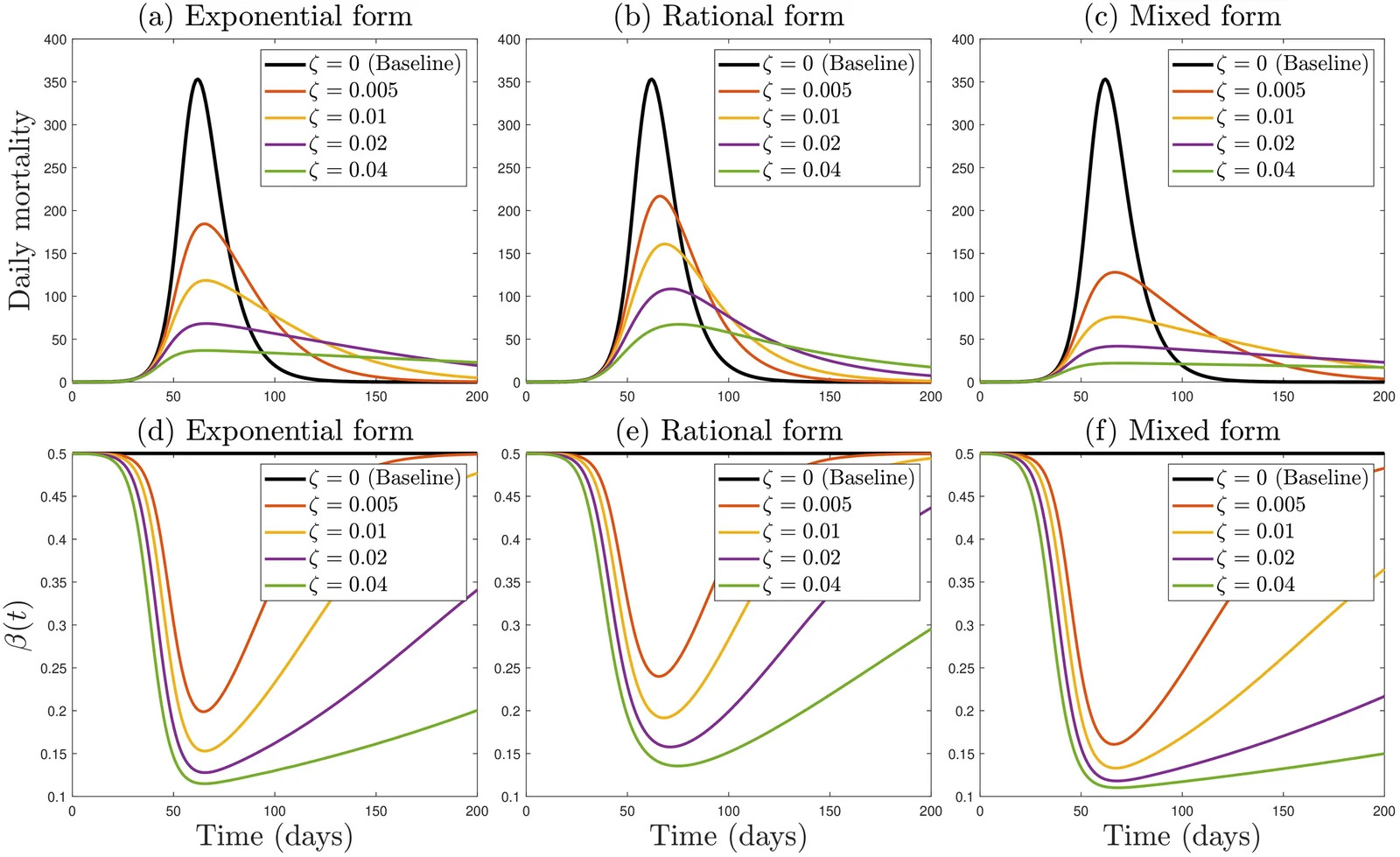
[Sensitivity of ζ in three behavioral models] Daily mortality curves (top row) and corresponding functional form of the transmission rates β(t) (bottom row) for three behavioral models with varying behavioral response strength ζ. (a) Exponential form: $\beta (t)=\beta_{0}e^{-\zeta \delta I(t)}$, where transmission rate decays exponentially with increasing infections. (b) Rational form: $\beta (t)=\beta_{0}/(1+\zeta \delta I(t))$, which saturates more slowly as infections rise. (c) Mixed form: $\beta (t)=\beta_{0}e^{-\zeta \delta I(t)}/(1+\zeta \delta I(t))$, combining both exponential and rational responses. Panels (d)–(f) show the time evolution of β(t) corresponding to panels (a)–(c), respectively. The baseline model (ζ=0, black line) represents traditional SEIRD dynamics without behavioral adaptation. Stronger behavioral responses (higher ζ values) lead to lower peak mortality, more prolonged epidemic tails, and greater sustained suppression of β(t). Model parameters: σ=1/3 day^-1^, γ=1/10 day^-1^, δ=0.001 day^-1^, $\beta_{0}=0.5$ day^-1^, *N* = 10^6^.

Fig 1 illustrates how the three functional forms respond to varying values of ζ. Across all forms, increasing ζ produces lower peak mortality and a more prolonged post-peak decline, while β(t) undergoes a sharp reduction during the mortality surge before gradually recovering as deaths subside. The forms differ in the degree and persistence of transmission suppression: as mortality rises and falls over the course of the outbreak, the mixed form attains the greatest reduction in β(t) most rapidly and recovers most slowly toward $\beta_{0}$, while the rational form produces the weakest suppression and recovers fastest. The exponential form is intermediate in both respects.

#### Daily mortality.

While individuals change behavior in response to instantaneous mortality δI, we compare model predictions to observed daily mortality data. Model-simulated daily mortality on day *t* is defined as:


$$\begin{array}{c} M_{\text{sim}}(t)=D(t)-D(t-1), \end{array}$$ (2.5)

representing the daily increment in cumulative deaths. The time series $(M_{\text{sim}}(t){)}_{t}$ is compared to observed mortality data to estimate model parameters (Section 2.3).

#### Basic reproduction number.

The *basic reproduction number*, defined as the average number of secondary infections produced by a typical infectious individual in a wholly susceptible population (i.e., S(0)≈N(0)), is given by:


$$\begin{array}{c} {ℛ}_{0}=\frac{\beta_{0}}{\gamma +\delta }. \end{array}$$ (2.6)

#### Effective reproduction number.

The *effective reproduction number*, denoted by ${ℛ}_{e}(t)$, is the average number of new infections generated by a typical infected individual at time *t* [22]. The effective reproduction numbers for the baseline model, ${ℛ}_{e}^{f}(t)$, and behavioral model, ${ℛ}_{e}^{b}(t)$, are given respec*t*ively by:


$$\begin{array}{c} {ℛ}_{e}^{f}(t)=\frac{\beta_{0}}{\gamma +\delta }\times \frac{S(t)}{N(t)}\;\text{and}\;{ℛ}_{e}^{b}(t)=\frac{\beta (t)}{\gamma +\delta }\times \frac{S(t)}{N(t)}. \end{array}$$ (2.7)

#### Final epidemic size.

The final epidemic size, denoted *A*, represents the total proportion of the population that becomes infected over the course of an outbreak:


$$\begin{array}{c} A=\frac{R(\infty )+D(\infty )}{N}, \end{array}$$ (2.8)

where R(∞) and D(∞) denote the cumulative recovered and deceased individuals as t→∞. Numerically, we compute *A* from simulated SEIRD trajectories by integrating until the infectious compartment falls below a threshold (*I*(*t*) < 1) and calculating $A=(N-S(t_{\text{end}}))/N$, where *t*_end_ is the time at which the epidemic effectively terminates.

### 2.2 Data and location selection

We calibrated the models to COVID-19 mortality data from the COVID-19 Data Repository by the Center for Systems Science and Engineering (CSSE) at Johns Hopkins University [23]. Our analysis focused on the first wave of the pandemic in the United States, covering the 123-day period from March 1, 2020 to July 1, 2020. This period is chosen because it preceded the emergence of major SARS-CoV-2 variants of concern [24] and the availability of vaccines and standardized antiviral treatments, with transmission dynamics driven primarily by the ancestral strain in a largely immunologically naive population, and because non-pharmaceutical interventions (NPIs) — including stay-at-home orders, school closures, and mask mandates — represented the primary public health response, making behavioral adaptation the dominant mechanism modulating transmission [6,7].

#### Location selection criteria.

From the 51 locations, we retained 20 for analysis by applying two exclusion criteria to the 7-day moving average of daily reported deaths, $y_{\ell }(t)$, for each location ℓ. The criteria were designed to ensure that each included trajectory carried a sufficient mortality signal for reliable model fitting and captured a sufficiently complete first mortality wave within the observation window. Here, a mortality wave refers to a mortality trajectory with an identifiable growth phase, a well-defined mortality peak, and a subsequent temporal decline within the observation window.

1. *Insufficient mortality signal.* Because reliable fitting of a deterministic model requires a sufficient mortality signal, we excluded locations whose total smoothed mortality signal over the analysis window, $\sum_{t}y_{\ell }(t)$, was below 50 deaths, or whose peak smoothed daily mortality, $\max_{t}y_{\ell }(t)$, did not reach 10 deaths per day.
2. *Incomplete mortality wave.* Because good parameter estimation requires the mortality trajectory to carry information about both epidemic growth and decline, we excluded locations for which the first mortality wave was not sufficiently resolved within the observation window (i.e., 123 days). Specifically, locations were excluded if the mortality peak occurred fewer than 21 days before the end of the observation window (this allows us to exclude locations where mortality is still growing during the observation window), or if the mean smoothed daily mortality over the final 14 days exceeded 40% of the peak (this allows us to exclude locations where mortality plateaus after peak).

Colorado (CO) was additionally excluded because of a reporting discontinuity in the cumulative mortality data that produced an artificial mortality spike.

The 20 retained locations are: CT, DC, DE, IA, IL, IN, LA, MA, MD, MI, MN, NJ, NM, NY, OH, PA, RI, VA, WA, and WI. Details on the 31 excluded locations and their mortality trajectories are provided in Fig A1 and Table A2 in S1 Appendix. Full location names and standard two-letter abbreviations for all 51 locations are given in Table A1 in S1 Appendix. As a sensitivity analysis, we also report model-comparison results across all 51 locations, including those excluded from the main analysis, for the mixed behavioral model in Table A3 in S1 Appendix; analogous sensitivity tables for the exponential and rational behavioral models are provided in Tables A4 and A5 in S1 Appendix. These sensitivity results are included to show how the main qualitative conclusions vary under relaxed inclusion criteria, but estimates from excluded locations should be interpreted cautiously because those trajectories fail one or more data screening criteria.

#### Data preprocessing.

We applied a 7-day moving average to daily mortality counts to reduce reporting artifacts.

#### Population data.

Initial location population sizes *N*(0) were obtained from the JHU CSSE dataset, ranging from 705,749 (DC) to 19,453,561 (NY).

### 2.3 Parameter inference

We infer parameters using Approximate Bayesian Computation with Sequential Monte Carlo (ABC–SMC) [25–27], as implemented in the pyABC library [28], with the deterministic SEIRD model (2.1). The complete codebase for simulation, inference, and figure generation is available at https://github.com/markolalovic/epi-behavior-models. Fixed and inferred parameters are summarized in Table 1; additional implementation details, including ABC–SMC stopping criteria, perturbation kernels, and tolerance schedules, are provided in Section A3 in S1 Appendix. For each location and model, we simulate daily deaths over the 123-day window and apply the same 7-day moving average used for the observed data.

**Table 1:** Fixed and inferred model parameters and initial conditions. Fixed parameters are set based on early SARS-CoV-2 data; inferred parameters are estimated via ABC–SMC separately for each location and model variant. 𝒰(a,b) denotes a continuous Uniform distribution on [*a*,*b*]; log-uniform priors place 𝒰 on the log scale.

Symbol	Description	Value / Prior	Source
σ	Incubation rate	1/3 day^-1^ (fixed)	[29,30]
γ	Recovery rate	1/10 day^-1^ (fixed)	[31,32]
${ℛ}_{0}$	Basic reproduction number	𝒰(1.2, 6.0)	Inferred
$\pi_{0}=I_{0}/N$	Initial prevalence	$\log\pi_{0}\sim 𝒰(\log10^{-8},\;\log10^{-3})$	Inferred
δ	Disease-induced mortality rate	$\log\delta \sim 𝒰(\log10^{-6},\;\log10^{-2})$	Inferred
ζ	Behavioral response strength	𝒰(0, 0.05)	Inferred
*E*_0_	Initial exposed	1 (fixed)	Assumed
*I*_0_	Initial infectious	$\max\{1,\;N\pi_{0}\}$	Calculated
*R*_0_	Initial recovered	0 (fixed)	Assumed
*D*_0_	Initial deceased	0 (fixed)	[23]
*S*_0_	Initial susceptible	$N-(E_{0}+I_{0}+R_{0}+D_{0})$	Calculated

We reparameterize in epidemiological terms, inferring ${ℛ}_{0}$, the initial prevalence $\pi_{0}=I_{0}/N$, the disease-induced mortality rate δ, and the behavioral response parameter ζ. (We note that $\beta_{0}$ is not directly inferred but can be calculated from the inferred ${ℛ}_{0}$ based on (2.6).) The prior on ${ℛ}_{0}$ is chosen to be weakly informative:


$$\begin{array}{c} {ℛ}_{0}\sim 𝒰(1.2,6.0). \end{array}$$

The lower bound excludes subcritical or nearly subcritical regimes that are inconsistent with the selected mortality trajectories, all of which exhibit a clear first mortality wave. The upper bound is deliberately conservative, allowing for uncertainty in early SARS-CoV-2 transmissibility and for the fact that, in behavioral models, ${ℛ}_{0}$ represents intrinsic transmissibility before behavioral adaptation reduces contacts.

The priors on the initial prevalence and mortality rate are log-uniform,


$$\begin{array}{c} \log\pi_{0}\sim 𝒰(\log10^{-8},\log10^{-3}),\;\log\delta \sim 𝒰(\log10^{-6},\log10^{-2}), \end{array}$$

because both parameters are positive, expected to be small, and uncertain over several orders of magnitude.

For the behavioral response strength we use


$$\begin{array}{c} \zeta \sim 𝒰(0,0.05). \end{array}$$

Parameter ζ is phenomenological rather than directly measured. Its scale is determined by the dimensionless product ζδI(t), where δI(t) is the instantaneous disease-induced mortality rate. Thus ζ controls the daily-mortality scale over which transmission is reduced. The prior includes ζ=0, corresponding to no behavioral feedback, and extends to values producing substantial reductions in transmission at mortality levels observed in the retained locations. For example, under the exponential form, ζ=0.05 gives transmission multipliers $e^{-0.5}\approx 0.61$, $e^{-2.5}\approx 0.08$, and $e^{-5}\approx 0.007$ at mortality rates of 10, 50, and 100 deaths per day, respectively. Therefore, the prior spans negligible, moderate, and strong behavioral responses.

To calibrate model-simulated to observed mortality trajectories, we use the *Normalized Sum of Squared Errors (NSSE):*


$$\begin{array}{c} d_{NSSE}(y^{sim},y^{obs})=\frac{\sum_{t=1}^{T}(y^{sim}(t)-y^{obs}(t){)}^{2}}{\sum_{t=1}^{T}y^{obs}(t{)}^{2}}, \end{array}$$ (2.9)

where $y^{sim}(t)$ is the model-simulated 7-day daily moving average mortality and $y^{obs}(t)$ is the observed 7-day moving average mortality over the *T* = 123 day observation window. This normalization ensures the distance is scale-invariant across locations with different mortality burdens.

ABC–SMC uses 1000 particles per generation, a multivariate normal transition kernel adapted from the previous population, and a quantile-based tolerance schedule over up to 10 generations. Additional details are provided in Section A3 in S1 Appendix.

### 2.4 Synthetic data experiments

To rigorously test whether behavioral effects can be recovered from mortality data and to quantify systematic biases in parameter estimation, we conducted controlled synthetic data experiments. We generated mortality trajectories from behavioral models using parameters calibrated to Massachusetts COVID-19 data, with varying values of the behavioral parameter ζ. To approximate observational variability, we added small independent Gaussian noise to each simulated series:


$$\begin{array}{c} M_{t}^{\text{obs}}=M_{t}+\varepsilon_{t},\;\varepsilon_{t}\sim 𝒩(0,\;\sigma^{2}),\;\sigma =0.025\max_{t}M_{t}. \end{array}$$

Negative values were truncated to zero before applying the 7-day moving average. We then fitted both the data-generating behavioral model and the baseline model to these synthetic mortality datasets. This approach allows us to assess: (i) whether the behavioral model correctly recovers the known underlying dynamics; (ii) whether the baseline model produces systematic biases when behavioral feedback is present but ignored; and (iii) how the magnitude of behavioral response (controlled by varying ζ) affects the ability to distinguish between model structures.

Note that it is expected by construction that the behavioral model recovers the synthetic data better than the baseline; the purpose of these experiments is not to demonstrate this, but rather to confirm that systematic biases in ${ℛ}_{0}$ and final epidemic size arise specifically from model misspecification. By knowing the true data-generating mechanism, we can definitively attribute any systematic biases in ${ℛ}_{0}$ and final epidemic size observed in the empirical data to model misspecification, rather than to data noise (e.g., stochastic fluctuations in daily death counts), reporting irregularities (e.g., batch corrections, delayed notifications, or inconsistent cause-of-death classification across states), or other surveillance heterogeneities inherent to COVID-19 mortality data.

### 2.5 Model comparison

We evaluate the relative performance of baseline and behavioral models using Bayesian model selection via ABC–SMC. We describe below the distance metric and Bayes factor calculation used for model comparison.

#### Distance metric.

We use the NSSE, given by Eq 2.9, as one of our model comparison metrics.

#### Bayes factor.

The Bayes factor is a measure of the relative evidence provided by the data in favor of one model over another, defined as the ratio of their marginal likelihoods [33]. Under equal model priors, it reduces to the posterior odds ratio. We performed ABC–SMC model selection [25–27] comparing the baseline model against each of the three behavioral model formulations (exponential, rational, and mixed) separately, assigning equal prior probabilities to each pair. To avoid extinction artifacts, we report results at the first stabilized generation $g^{*}$, defined as the earliest *g* for which both model probabilities change by less than 0.02 over 3 consecutive *g*enerations and P(Baseline∣D,g)>0. At the stabilized generation $g^{*}$, we summarize the evidence for a behavioral model via the Bayes factor under equal model priors:


$$\begin{array}{c} {\text{BF}}_{\text{eq}}=\frac{P(M_{\text{Behavior}}|D,g^{*})}{P(M_{\text{Baseline}}|D,g^{*})}. \end{array}$$

We interpret BF_eq_ following the scale from [33]. Values of BF_eq_ > 1 indicate evidence favoring the behavioral model over the baseline model, with strength categorized as: 1–3 (*very weak*), 3–20 (*positive*), 20–150 (*strong*), and > 150 (*very strong*). Values < 1 indicate evidence favoring the baseline model.

## 3. Results

We fitted COVID-19 mortality data from 20 US locations (19 U.S. states and the District of Columbia) during the first pandemic wave (March–July 2020) to a baseline SEIRD model with constant transmission rate and three behavioral SEIRD models where transmission rate declines in response to disease-induced mortality. The 20 retained locations were selected using pre-specified data-quality criteria designed to isolate clear first-wave mortality trajectories suitable for single-wave inference (see Section 2 for detailed selection criteria). Our analysis reveals three key findings: (i) behavioral models provide significantly improved fits to observed mortality data across almost all locations examined, with both median normalized squared error and Bayes factors favoring behavioral models in 18 of 20 locations; (ii) baseline models that ignore behavioral change systematically underestimate the basic reproduction number (${ℛ}_{0}$), with median ${ℛ}_{0}$ from behavioral models consistently higher than from baseline models across all locations; and (iii) baseline models overestimate total infection burden despite underestimating transmissibility, creating a paradoxical dual bias with serious policy implications.

### 3.1 Behavioral models show superior fit to COVID-19 mortality data

**Fig 2:**
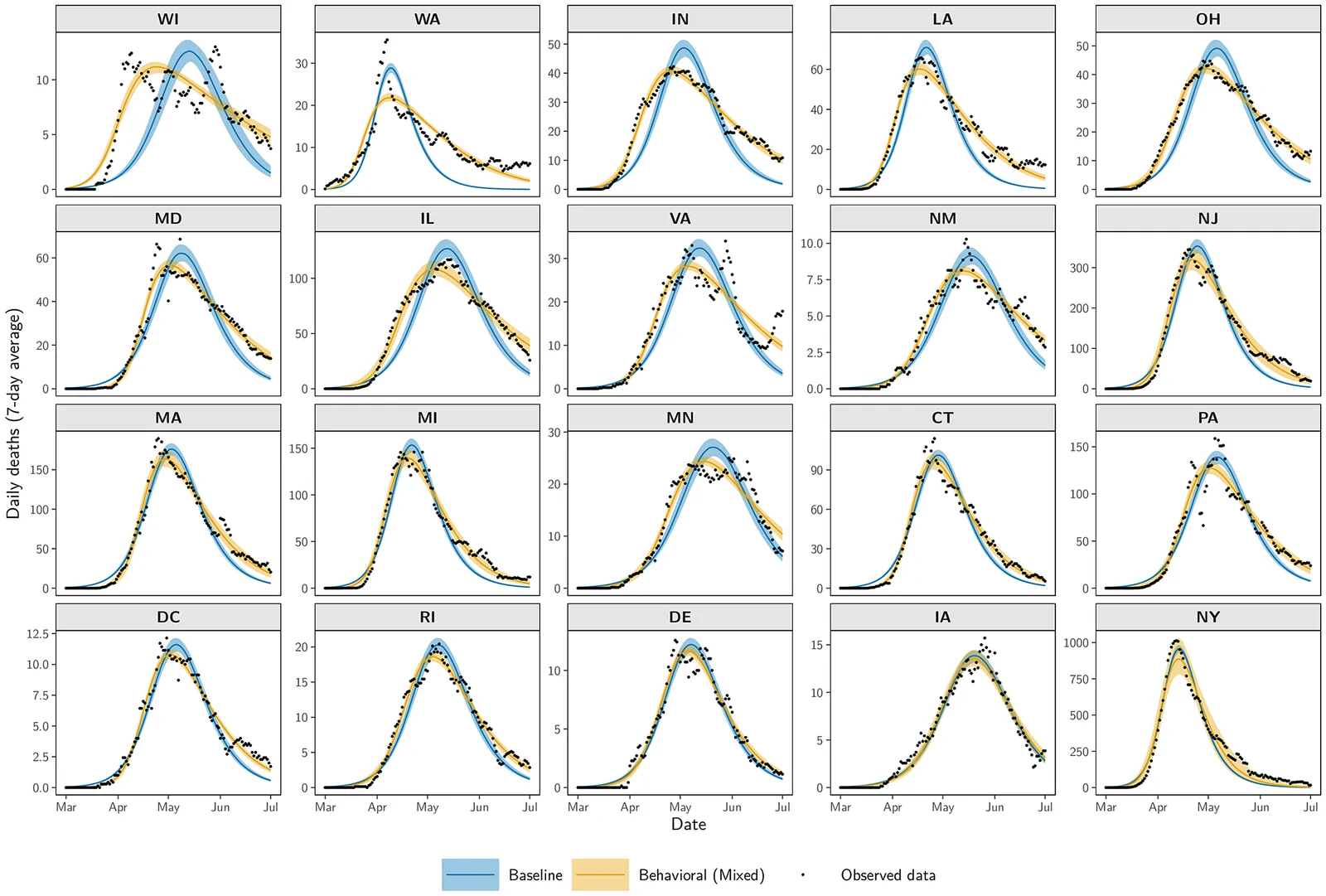
Behavioral models capture observed mortality dynamics better than baseline models across 20 US locations. Posterior predictive fits comparing baseline and behavioral (mixed) models to 7-day averaged daily COVID-19 deaths during the first pandemic wave (March–July 2020). Black points show observed mortality data. Solid lines show posterior predictive medians and shaded regions show 90% credible intervals (5th–95th percentiles), computed from 1,000 weighted posterior samples drawn from the final ABC–SMC generation. The behavioral model accurately captures the characteristic asymmetric mortality curves with sharp initial rises and gradual extended declines observed across most locations. Locations are ordered from left to right and top to bottom by decreasing improvement in fit: those where the behavioral model outperforms the baseline model most dramatically (largest difference in median NSSE) appear first, progressing to locations where the two models perform most similarly. This ordering visually emphasizes that behavioral feedback provides substantial improvements in model fit across the vast majority of locations examined.

All three behavioral models (exponential, rational, and mixed) consistently outperformed the baseline model when fitted to COVID-19 mortality data from 20 locations during the first pandemic wave. Visually, the behavioral models capture the characteristic asymmetric mortality curves observed in many locations—sharp initial rises followed by gradual, extended declines—while the baseline model produces curves that decay too rapidly (Fig 2 shows the mixed behavioral model; Figs A4 and A5 in S1 Appendix show exponential and rational formulations, respectively).

This superior performance is confirmed quantitatively across all three formulations. For the mixed behavioral model, we observed lower median normalized sum of squared errors (NSSE) in 18 of 20 locations, with Bayesian model selection via ABC–SMC providing evidence for the mixed behavioral model in these same 18 locations (Table 2). Only Iowa and New York favored the baseline model. The exponential and rational formulations of behavioral models demonstrated comparable performance (see Tables A6 and A7 in S1 Appendix). Overall, across all three behavioral formulations, at least 18 of 20 locations favored behavioral models over baseline models based on NSSE and Bayes factor.

**Table 2:** Comparison of the baseline model and the behavioral (mixed) model across 20 US locations. For each location, Median NSSE_B_ and Median NSSE_M_ denote the median posterior normalized sum of squared errors (NSSE) under the baseline *B* and behavioral (mixed) *M* models, respectively. BF_eq_ is the Bayes factor under equal model priors in favor of the mixed model versus the baseline model at the stabilized ABC–SMC generation, and “Evidence” gives the corresponding verbal strength of evidence. Table is ordered by decreasing Bayes factor in favor of the behavioral model. See Section 2 for interpretation guidelines for BF_eq_.

Location	Median NSSE_B_	Median NSSE_M_	BF_eq_	Evidence
DC	0.0286	**0.0133**	11551.24	very strong
RI	0.0251	**0.0107**	852.43	very strong
NM	0.0493	**0.0151**	688.87	very strong
IN	0.0900	**0.0087**	126.43	strong
CT	0.0331	**0.0121**	121.80	strong
WI	0.2540	**0.0559**	105.04	strong
VA	0.0815	**0.0388**	100.58	strong
MN	0.0398	**0.0185**	79.31	strong
WA	0.1784	**0.0656**	64.52	strong
OH	0.0685	**0.0058**	37.48	strong
IL	0.0574	**0.0118**	35.61	strong
MA	0.0377	**0.0143**	22.96	strong
PA	0.0472	**0.0311**	19.86	positive
LA	0.0837	**0.0166**	13.46	positive
MD	0.0617	**0.0152**	11.97	positive
MI	0.0345	**0.0123**	11.80	positive
DE	0.0214	**0.0167**	10.67	positive
NJ	0.0430	**0.0149**	3.67	positive
IA	**0.0139**	0.0168	0.51	favors Baseline
NY	**0.0277**	0.0310	0.02	favors Baseline

Among the three behavioral formulations, the mixed model most consistently outperformed the baseline, particularly in locations with sharp, high-intensity mortality peaks such as DC. This likely reflects the mixed form’s faster saturation at high mortality levels (as depicted in Fig 1) - a property absent in the exponential and rational forms individually. In locations with more gradual mortality trajectories, the three forms produced comparable performance, suggesting that mortality signal strength mediates which functional form is most identifiable from data.

The superior performance of behavioral models varies systematically across locations, with the largest improvements in locations exhibiting extended mortality decline phases. Fig 2 orders locations by decreasing difference in median NSSE between baseline and behavioral models, with locations showing the largest improvement appearing first. Locations like Washington and Indiana (top left) exhibit the most striking contrast—the behavioral models capture gradual, extended mortality declines that baseline models fail to reproduce. Moving toward Rhode Island and Delaware (bottom right), the performance gap narrows, with both model types achieving similar fit quality.

Sensitivity analysis reveals a key feature of behavioral models: increasing the behavioral response parameter (ζ) produces lower peak mortality and slower decline in daily deaths following the peak (Fig 1), a mechanism absent in baseline models. This pattern is reminiscent of the “flattening the curve” phenomenon, where public health interventions reduce peak mortality while extending the duration of the outbreak. In all three behavioral formulations, β(t) exhibits a characteristic pattern: sharp decline during the mortality surge followed by gradual recovery as deaths decline (see Section A6 in S1 Appendix). This time-varying transmission rate better reflects the reality of behavioral adaptation during epidemics, consequently producing superior fits to observed mortality data compared to the constant transmission rate assumption of the baseline model.

We validated this finding through controlled synthetic data experiments where mortality trajectories were generated from behavioral models with varying levels of behavioral sensitivity (ζ), then fitted to both the data-generating behavioral model and the baseline model. Using the mixed behavioral formulation, behavioral models accurately recovered the true dynamics across all ζ values, while the baseline model increasingly failed to capture mortality trajectories as behavioral feedback strengthened (Fig 4A). Results were consistent across all three functional forms examined (Figs A2A and A3A show exponential and rational forms in S1 Appendix, respectively), demonstrating that baseline models systematically mischaracterize epidemic trajectories when behavioral responses are present.

### 3.2 Baseline models systematically underestimate the basic reproduction number

**Fig 3:**
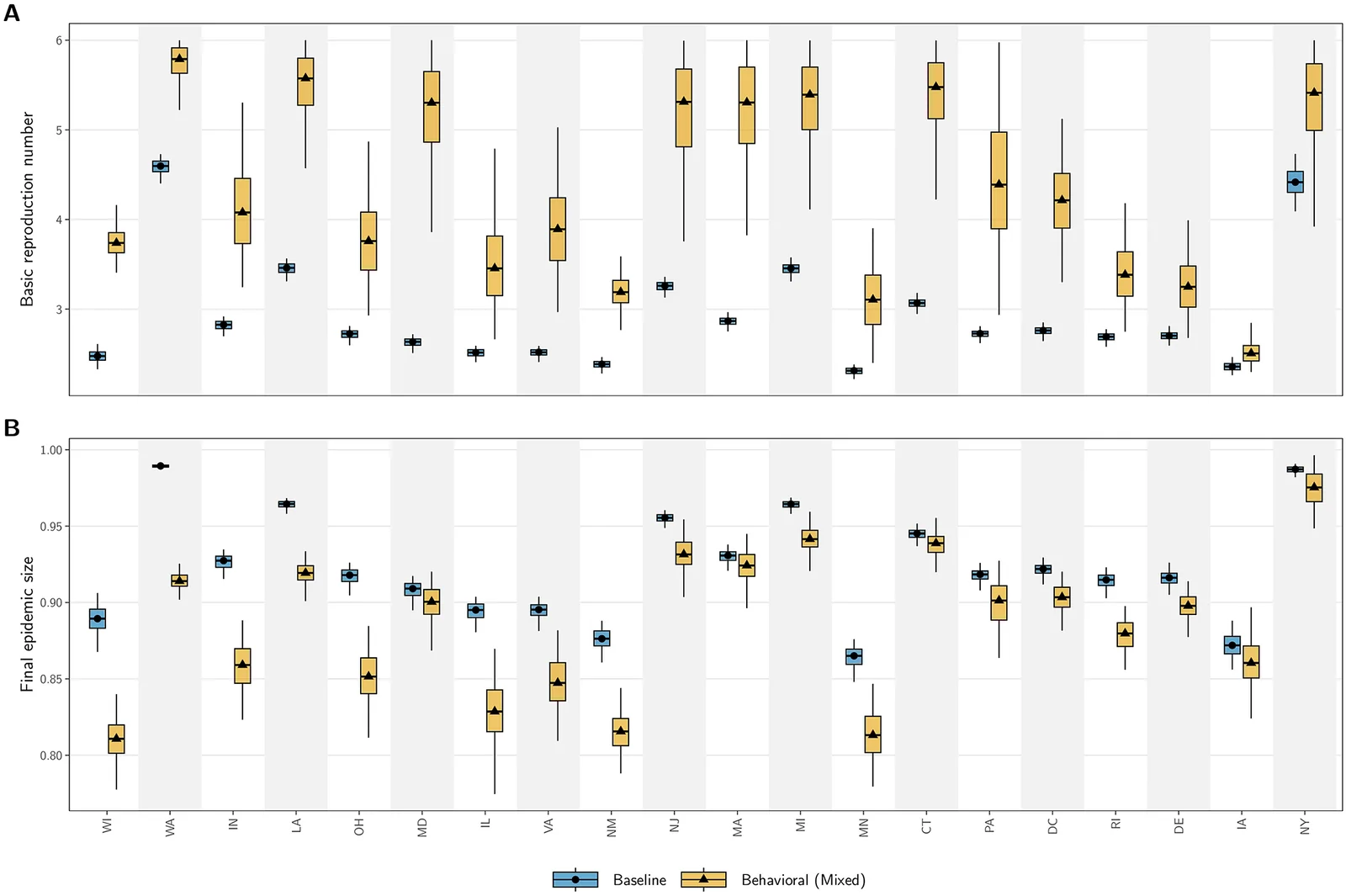
Baseline models systematically underestimate basic reproduction number while overestimating final epidemic size across all 20 locations. Posterior distributions of key epidemiological quantities estimated by fitting the baseline and behavioral (mixed) models to COVID-19 mortality data from 20 US locations during the first pandemic wave (March–July 2020). (A) Basic reproduction number ${ℛ}_{0}$. The behavioral model consistently estimates higher ${ℛ}_{0}$ values than the baseline model across all 20 locations, indicating that models ignoring behavioral feedback systematically underestimate intrinsic transmissibility. (B) Final epidemic size (proportion of population infected). Despite estimating lower ${ℛ}_{0}$, the baseline model predicts larger cumulative infection burdens than the behavioral model in all 20 locations, demonstrating the paradoxical consequence of ignoring behavioral adaptation. Boxplots show median (center line), interquartile range (box), and whiskers extending to 1.5×IQR within the sample support computed from weighted posterior samples. Locations are ordered from left to right by decreasing difference in median NSSE between the baseline and behavioral (Mixed) models.

Baseline models that ignore behavioral change systematically underestimate the basic reproduction number (${ℛ}_{0}$) compared to behavioral models when fitted to the same COVID-19 mortality data across the 20 selected US locations (Fig 3A shows the comparison with the mixed formulation). For the behavioral mixed model, the baseline model inferred a lower median ${ℛ}_{0}$ in all 20 retained locations. The cross-location difference, defined as $\mathrm{median}({ℛ}_{0}^{M})-\mathrm{median}({ℛ}_{0}^{B})$, had median 1.26, interquartile range 0.91–1.97, and range 0.15–2.67. Furthermore, ${ℛ}_{0}$ estimates from all three behavioral model variants were similar to each other, and the systematic underestimation by the baseline model was consistent across the exponential, rational, and mixed formulations (Figs A6 and A7 in S1 Appendix show ${ℛ}_{0}$ comparisons for the exponential and rational forms, respectively).

The effective reproduction number for all four models is provided in Section A7 in S1 Appendix. In all cases, ${ℛ}_{e}(t)$ converges to values below 1 by late April–May across all locations (coinciding with peak infection), though behavioral models exhibit steeper initial declines corresponding to rapid behavioral responses during the early stages of the outbreak.

**Fig 4:**
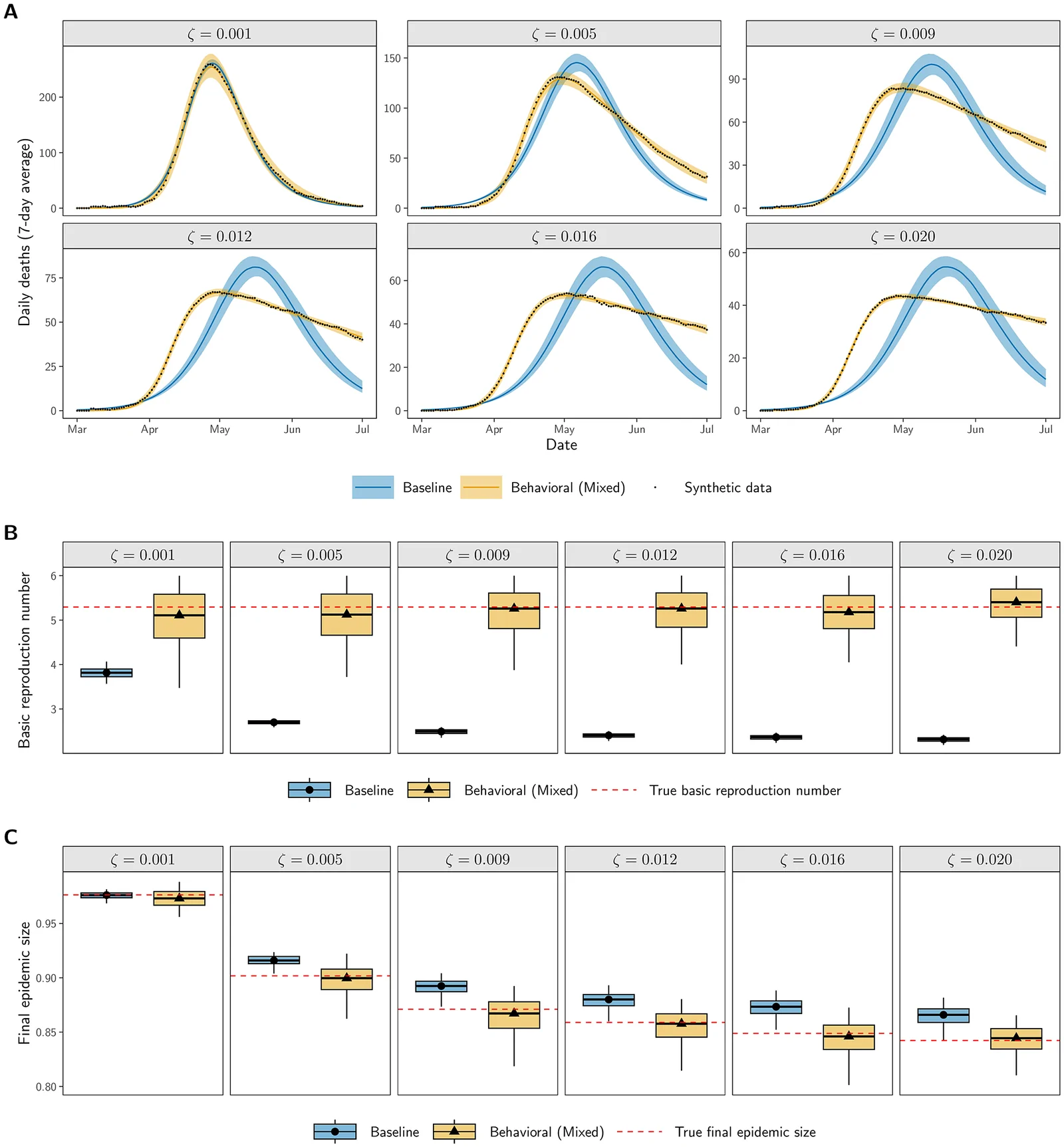
Baseline models systematically misestimate epidemic quantities when behavioral feedback is present but ignored. Synthetic data experiments where mortality trajectories were generated using the behavioral (mixed) model with known parameters and varying behavioral sensitivity ζ, then fitted with both baseline and behavioral models. **(A)** Posterior predictive fits to synthetic mortality data (black points). Solid lines show posterior medians and shaded regions show 90% credible intervals. The behavioral (mixed) model accurately recovers the true dynamics across all ζ values, while the baseline model increasingly fails to capture the extended post-peak decline as behavioral feedback strengthens with increasing ζ values. **(B)** Posterior distributions of the basic reproduction number ${ℛ}_{0}$. The behavioral model correctly recovers the true ${ℛ}_{0}$ (red dashed line) across all scenarios, while the baseline model systematically underestimates ${ℛ}_{0}$, with bias increasing as ζ increases. **(C)** Posterior distributions of final epidemic size. The behavioral model accurately estimates the true final size (red dashed line), while the baseline model systematically overestimates infection burden despite underestimating transmissibility.

Synthetic experiments where true ${ℛ}_{0}$ was known by design confirmed this systematic underestimation. Baseline models consistently underestimated true ${ℛ}_{0}$ across all behavioral sensitivity values, with bias increasing as behavioral feedback strengthened (increasing ζ), while behavioral models accurately recovered true ${ℛ}_{0}$ across all ζ values (Fig 4B for mixed; Figs A2B and A3B for exponential and rational forms in S1 Appendix, respectively). This confirms that ${ℛ}_{0}$ underestimation is a systematic consequence of model misspecification.

Intuitively, this systematic underestimation of ${ℛ}_{0}$ arises because baseline and behavioral models, although fitted to the same observed mortality data, assume fundamentally different transmission mechanisms. The baseline model uses a constant transmission rate $\beta_{0}$ throughout the epidemic, fitting the data with a moderate value that remains fixed. In contrast, behavioral models assume a time-varying transmission rate β(t) that declines as people adapt their behavior in response to rising mortality. To reproduce the same initial epidemic growth observed in the data, behavioral models require a substantially higher initial transmission rate $\beta_{0}$—which then declines as behavioral responses take effect. As evident from Eq 2.6, ${ℛ}_{0}$ is an increasing function of $\beta_{0}$; hence models that estimate higher values of $\beta_{0}$ necessarily yield higher estimates of ${ℛ}_{0}$. Thus, behavioral models, which require higher $\beta_{0}$ to account for subsequent behavioral adaptation, naturally produce higher ${ℛ}_{0}$ estimates than baseline models. This higher initial transmission rate captured by behavioral models represents the intrinsic transmissibility of the pathogen before any behavioral adaptation occurs. The baseline model, lacking this behavioral feedback mechanism, systematically underestimates this initial transmissibility because it interprets the entire epidemic trajectory—including periods when behavior has already adapted—through the lens of a single, constant transmission rate.

### 3.3 Ignoring behavioral responses overestimates infection burden

Despite underestimating ${ℛ}_{0}$, baseline models overestimate the total proportion of the population that becomes infected (Fig 3B shows the comparison with the mixed formulation; Figs A6B and A7B in S1 Appendix show comparisons with exponential and rational forms, respectively). For the behavioral mixed model, the baseline model produced a larger median final-size estimate in all 20 retained locations. The cross-location overestimation, defined as $\mathrm{median}(A^{B})-\mathrm{median}(A^{M})$, had median 2.96 percentage points, interquartile range 1.58–6.22 percentage points, and range 0.64–7.87 percentage points. This overestimation was largest in WI, where the baseline model predicted a median final epidemic size that was 7.87 percentage points higher than the behavioral mixed model.

Synthetic experiments confirmed this systematic overestimation of final epidemic size. Baseline models consistently overestimated true final epidemic sizes while behavioral models accurately recovered them across all ζ values considered (Fig 4C shows comparison with mixed; Figs A2C and A3C show comparisons with exponential and rational forms in S1 Appendix, respectively). The bias in overestimating final epidemic size increases with stronger behavioral responses, demonstrating that ignoring behavioral dynamics leads to inflated infection burden estimates regardless of functional form.

To gain theoretical insight into the relationship between behavioral feedback and epidemic outcomes, we analytically examine final epidemic size under a controlled scenario where ${ℛ}_{0}$ is identical for both the baseline and a behavioral model. For analytical tractability, we assume a constant total population *N* = *S* + *E* + *I* + *R* + *D*, which differs from the time-varying living population N(t)=N(0)−D(t) used in our numerical simulations. We prove that for any fixed value of ${ℛ}_{0}$, behavioral models incorporating mortality-driven feedback produce final epidemic sizes less than or equal to those from baseline models. We first demonstrate this result for the exponential behavioral model.

**Theorem 3.1 (Final Size Reduction: Exponential Form).** *Consider an epidemic model with behavioral response function* $\beta (t)=\beta_{0}\exp(-\alpha I(t))$ *where* α=ζδ*. Furthermore, we assume that the total population size is fixed with N = S + E + I + R + D. Let A*_*0*_ *denote the final size of the baseline model (i.e.,* α=0*). The final size of the behavioral model,* $A_{\alpha }$*, can be expressed as:*


$$\begin{array}{c} A_{\alpha }=A_{0}+\alpha a_{1}+O(\alpha^{2}), \end{array}$$

*where a_1_ < 0 is the first-order correction to the final epidemic size due to behavioral feedback. Hence, for small* α*, the final size of the behavioral model is smaller than that of the baseline model.*

The proof of Theorem 3.1, based on asymptotic expansion, is given in Appendix A.

This result extends beyond the exponential form. For any behavioral response function satisfying $0<\beta (t)\leq \beta_{0}$—including the exponential, rational, and mixed forms considered in this study—we show that behavioral models always produce smaller or equal final epidemic sizes compared to baseline models when ${ℛ}_{0}$ is the same for both models.

**Theorem 3.2 (Final Size Reduction: General Form).** *Consider the SEIRD model (2.1) where N = S + E + I + R + D is the fixed total population size and the transmission rate* $\beta_{\alpha }(t)=\beta_{0}\Psi (\alpha ,I_{\alpha }(t))$ *decreases with increasing mortality through a behavioral response function satisfying* $0<\Psi (\alpha ,I_{\alpha }(t))\leq 1$*, with* $\beta_{0}$ *being the maximum transmission rate and* α=ζδ*. If a behavioral model (with* α>0*) and the baseline model (with* α=0 *and constant transmission* $\beta_{0}$*) have equal basic reproduction numbers, given by* ${ℛ}_{0}=\beta_{0}/(\gamma +\delta )$*, then the final epidemic size of the baseline model (A*_*0*_*) is always larger than or equal to that of the behavioral model (*$A_{\alpha }$*).*

The proof of Theorem 3.2 is given in Appendix B. We obtain final size relationships for the baseline model with constant transmission rate $\beta_{0}$ and for behavioral models with $\beta (t)<\beta_{0}$. By defining an auxiliary function based on these final size equations and exploiting its properties, including its unique maximum and concavity, we show that the final size of the behavioral model is always less than or equal to that of the baseline model when both share the same basic reproduction number ${ℛ}_{0}$.

## 4. Discussion

Our analysis of COVID-19 mortality data from 20 US locations during the first pandemic wave reveals systematic biases that arise when epidemic models fail to account for behavioral responses to disease burden. Across all three behavioral models, at least 18 of 20 locations favored behavioral models—where transmission rates decline as mortality increases—over the traditional model with a constant transmission rate, based on both NSSE and Bayes factor. More critically, we identified a problematic paradox: models that ignore behavioral change systematically underestimate pathogen transmissibility while simultaneously overestimating total infection burden. We confirmed these biases through controlled synthetic experiments where the true dynamics were known by design, demonstrating that the discrepancies arise from model misspecification rather than data quality or stochastic effects. These findings held across three distinct mathematical formulations of behavioral models, strengthening confidence in our conclusions.

The dual bias we identify has potential implications for public health decision-making. Models that ignore behavioral responses underestimate transmissibility as measured by the basic reproduction number, which may contribute to delayed or insufficiently aggressive early interventions. Conversely, overestimation of total infection burden could, in principle, contribute to excessive resource allocation for later epidemic phases that behavioral adaptation may prevent. While we do not formally evaluate allocation strategies or costs, these considerations suggest that the structural biases identified here are worth accounting for when translating model projections into policy — a direction that warrants dedicated future investigation.

The mechanism underlying these biases is intuitive once recognized. Baseline models with constant transmission rates fit observed mortality data using relatively modest transmission parameters because the transmission rate remains fixed throughout the inferred mortality wave. In contrast, behavioral models require substantially higher initial transmission rates to fit the same mortality data precisely because transmission declines as mortality increases and people adapt their behavior. This difference directly manifests in the basic reproduction number: the baseline model settles on a lower value that suffices under constant transmission, while the behavioral model correctly identifies the higher intrinsic transmissibility that characterized the pathogen before behavioral responses took effect. The bias in final epidemic size follows naturally from this same mechanism. When populations reduce contact rates in response to rising deaths—through actions like avoiding crowds, wearing masks, or increasing hygiene—fewer people ultimately become infected compared to scenarios where behavior remains unchanged.

This study has several important limitations. First, our models assume that behavioral changes are driven exclusively by location-specific reported mortality, whereas behavior in reality may also respond to mortality signals from nearby regions, national trends, policy announcements, or media coverage [6]. In addition, reported COVID-19 mortality may vary across locations because of differences in notification timing, testing availability, and cause-of-death classification, including the possibility of unrecognized COVID-19 deaths recorded under other causes [34]. Second, using mortality as a proxy for public risk perception does not capture the complex array of influences on behavior, including policy actions, misinformation, informal information channels, and individual risk tolerance [15]. This simplification may also overlook delays between changes in actual risk and behavioral adaptation, and neglects the possibility that current behavior may be influenced by past epidemic conditions (such as cumulative mortality over a preceding time window), neither of which our instantaneous mortality-driven feedback formulation captures [35,36]. Compartmental frameworks that explicitly encode richer behavioral determinants — including perceived susceptibility, perceived severity, fatigue — offer more mechanistic representations of behavioral adaptation [8,37], and comparing their performance against simpler phenomenological feedback formulations such as ours represents a valuable direction for future work.

Third, we assume homogeneous populations within each location, ignoring substantial variation in risk tolerance, contact patterns, and protective behavior adoption across age groups, socioeconomic strata, and communities. Real populations exhibit heterogeneous responses to epidemic threats, with some groups adopting protective measures rapidly while others respond more slowly or not at all [6,35,36,38]. Fourth, our analysis focuses exclusively on the first pandemic wave (March–July 2020), when behavioral responses were likely strongest due to novelty and uncertainty. Behavioral patterns may differ substantially in later waves as pandemic fatigue sets in or as populations develop more nuanced risk assessments [6]. We note further that behavioral suppression of an initial wave may leave a larger susceptible population, potentially contributing to epidemic resurgence in subsequent waves — a dynamic not captured by our single-wave framework.

Fifth, our models imply that extended post-peak mortality declines reflect stronger behavioral responses, but this relationship does not hold universally. New York experienced one of the earliest and most severe outbreaks [39–41] with stringent public health measures and well-documented behavioral changes [6,42,43], yet the mortality curve rose and fell relatively symmetrically without the prolonged tail our models associate with strong behavioral feedback. This disconnect suggests that the temporal signature of behavioral adaptation depends on factors we do not explicitly capture, such as the timing and stringency of policy interventions, underlying contact network structures, population density, or the rapidity with which protective behaviors were adopted. More broadly, our mortality-driven behavioral feedback implicitly aggregates multiple drivers of behavior change, including voluntary risk-aversion and policy-induced compliance, and cannot distinguish between these mechanisms at the aggregate level. Locations with sharp mortality declines might reflect either weak behavioral responses or, conversely, such rapid behavioral adaptation that transmission was quickly suppressed. Our models cannot distinguish between these possibilities, which limits our ability to infer behavioral mechanisms from mortality data alone. Finally, our behavioral models assume a specific functional relationship between mortality and transmission reduction. While we tested three different functional forms, real behavioral responses may follow more complex patterns involving thresholds, saturation effects, or time-varying sensitivity to disease burden. Relatedly, this study does not consider comparisons between baseline and behavioral models where the two differ in complexity by more than a single additional parameter. This is because having even a single additional parameter allows the model an additional degree of freedom to fit the data; hence, it is not unexpected that the behavioral model typically fits the data better than the baseline model, which can be obtained as a special case by turning off the behavioral response (i.e., setting ζ=0). Furthermore, even within the class of models considered here, the sensitivity of our qualitative and quantitative findings to the fixed epidemiological parameters remains unexplored. Because this study focuses on COVID-19, for which the incubation and infectious periods are relatively well characterized, we fixed both parameters at literature-informed values. Testing the sensitivity of our qualitative and quantitative findings to alternative values of the incubation and infectious periods is therefore a natural direction for future work, alongside testing sensitivity to different functional forms of behavioral feedback and to models of varying structural complexity.

Future work should address these limitations through several avenues. Incorporating spatial coupling between locations could capture how mortality in nearby regions influences local behavior [44,45]. Integrating data on policy interventions, mobility patterns, and direct behavioral surveys could provide more nuanced representations of behavioral change beyond mortality-driven responses. In particular, incorporating data on policy stringency — for example, from the Oxford COVID-19 Government Response Tracker [46] — alongside mortality signals could help disentangle the contributions of voluntary risk-aversion and policy-induced compliance to the behavioral signal recovered from empirical data. While age stratification is absent from our framework — with all variability in disease severity absorbed into the fitted parameter δ, this is a deliberate modeling choice: the homogeneous mixing assumption affords the mathematical tractability required to derive the analytical results of Theorems 3.1 and 3.2, and ensures that observed biases can be cleanly attributed to behavioral misspecification rather than confounded by heterogeneous age-dependent mortality, contact patterns, or behavioral responses. Age-structured models could account for heterogeneous contact patterns, risk perceptions, recovery rates, and disease severity across demographic groups [22]. As such, future studies should explore the effects of including an age-dependent infection fatality ratio (IFR) inference strategy as it has been well-documented that COVID-19 infection fatality ratios are known to depend heavily on age. Multi-wave analyses could reveal how behavioral responses evolve over the course of a pandemic as novelty effects diminish and risk perceptions stabilize. Comparing fits to multiple data streams—cases, hospitalizations, and deaths—could help disentangle behavioral responses to different epidemic indicators and their respective time lags. More fundamentally, developing frameworks that explicitly model the cognitive and social processes underlying behavioral change—such as risk perception dynamics, information diffusion, and social norm formation—could provide deeper mechanistic understanding beyond phenomenological feedback functions [15,47–50]. Such frameworks could better predict behavioral responses to novel pathogens and inform more effective risk communication strategies. Finally, the infectious period (10 days) and incubation period (3 days) are held fixed and identical across all models; while the latter differs from some published estimates for ancestral SARS-CoV-2, this choice ensures that differences in model performance are attributable solely to the presence or absence of behavioral feedback rather than to differences in assumed epidemiological parameters, and a comprehensive sensitivity analysis with respect to these quantities — including the choice of distributional assumptions for infectious periods — remains an interesting direction for future work.

Severing the coupling between pathogen transmission and human behavior produces models that systematically misrepresent reality. Infectious disease outbreaks unfold through feedback: transmission shapes behavior, and behavior alters transmission. Models that assume constant contact rates sacrifice this coupling for mathematical convenience, producing contradictory estimates of key epidemiological quantities—underestimating pathogen transmissibility while overestimating infection burden. These structural failures cascade through every decision that relies on model projections, from intervention timing to resource allocation.

Our findings demonstrate that incorporating behavioral dynamics is not merely a modeling refinement but essential for accurate epidemic parameter estimation and evidence-based pandemic response. Across 20 geographically and demographically diverse US locations, behavioral models consistently provided superior fits to observed data and more plausible estimates of both transmissibility and final epidemic size. The analytical results further establish that this dual bias is an inevitable mathematical consequence of ignoring behavioral feedback when it is present in the data. Future pandemic preparedness must prioritize the development and deployment of models that explicitly account for the dynamic interplay between disease transmission and human behavioral responses.

### A Proof of Theorem 3.1

*Proof.* Let $\beta_{\alpha }(t)=\beta_{0}\exp(-\alpha I_{\alpha }(t))$, where α=ζδ combines the behavioral sensitivity parameter ζ with the mortality rate δ, and $I_{\alpha }(t)$ denotes the infectious population trajectory corresponding to α. Furthermore, we assume that the total population size is fixed with *N* = *S* + *E* + *I* + *R* + *D*.

The *final epidemic size* corresponding to α is defined as


$$\begin{array}{c} A_{\alpha }:=R(\infty )+D(\infty )=(\gamma +\delta )\int_{0}^{\infty }I_{\alpha }(t)\;dt. \end{array}$$ (A.1)

From the *S*–equation of model (2.1), we have


$$\begin{array}{c} \frac{dS}{dt}=-\frac{\beta_{\alpha }(t)}{N}S(t)I_{\alpha }(t). \end{array}$$

Dividing both sides by *S*(*t*) and integrating in time from 0 to ∞ (noting *S*(*t*) > 0 for all finite time until ex*t*inction):


$$\begin{array}{c} \ln\left(\frac{S(\infty )}{S(0)}\right)=-\frac{1}{N}\int_{0}^{\infty }\beta_{\alpha }(t)I_{\alpha }(t)\;dt. \end{array}$$ (A.2)

Since the removed individuals at infinity equal the initial population minus susceptibles at infinity, we have


$$\begin{array}{c} S(\infty )=N-A_{\alpha },\;S(0)=S_{0}, \end{array}$$

and hence, using $\beta_{\alpha }(t)=\beta_{0}\exp(-\alpha I_{\alpha }(t))$, (A.2) can be rewritten as


$$\begin{array}{c} \ln\left(\frac{N-A_{\alpha }}{S_{0}}\right)=-\frac{\beta_{0}}{N}\int_{0}^{\infty }e^{-\alpha I_{\alpha }(t)}I_{\alpha }(t)\;dt. \end{array}$$ (A.3)

This holds for all α≥0. When α=0 (i.e., $\beta (t)\equiv \beta_{0}$), we have


$$\begin{array}{c} \ln\left(\frac{N-A_{0}}{S_{0}}\right)=-\frac{\beta_{0}}{N}\int_{0}^{\infty }I_{0}(t)\;dt=-\frac{\beta_{0}}{N(\gamma +\delta )}A_{0}, \end{array}$$

with *A*_0_ and *I*_0_(*t*) denoting the final size and infectious population corresponding to the baseline model. Hence, the classical implicit final-size equation:


$$\begin{array}{c} \ln\left(\frac{N-A_{0}}{S_{0}}\right)=-\frac{\beta_{0}}{N(\gamma +\delta )}A_{0}. \end{array}$$ (A.4)

#### Small-α expansion.

We now expand for small α>0. Let


$$\begin{array}{c} A_{\alpha }=A_{0}+\alpha a_{1}+O(\alpha^{2}),\;I_{\alpha }(t)=I_{0}(t)+\alpha J(t)+O(\alpha^{2}), \end{array}$$ (A.5)

where, *a*_1_ and *J*(*t*) are the first-order corrections to the final epidemic size and infectious trajectory, respectively.

We expand both sides of (A.3) using the Taylor series.

#### Left-hand side expansion.

We expand $\ln\left(\frac{N-A_{\alpha }}{S_{0}}\right)$ using Taylor series around *A* = *A*_0_:


$$\begin{array}{cc} \ln\left(\frac{N-A_{\alpha }}{S_{0}}\right) & =\ln\left(\frac{N-A_{0}}{S_{0}}\right)+\frac{d}{dA}\ln\frac{N-A}{S_{0}}{|}_{A_{0}}(A_{\alpha }-A_{0})+O((A_{\alpha }-A_{0}{)}^{2})\\ & =\ln\left(\frac{N-A_{0}}{S_{0}}\right)+{\frac{d}{dA}\ln\frac{N-A}{S_{0}}|}_{A_{0}}(\alpha a_{1})+O(\alpha^{2})\\ & =\ln\left(\frac{N-A_{0}}{S_{0}}\right)-\frac{\alpha a_{1}}{N-A_{0}}+O(\alpha^{2}). \end{array}$$ (A.6)

#### Right-hand side expansion.

Using $e^{-\alpha I_{0}}=1-\alpha I_{0}+O(\alpha^{2})$ and $I_{\alpha }$ from (A.5), we expand:


$$\begin{array}{c} e^{-\alpha I_{\alpha }}I_{\alpha }=(1-\alpha I_{0}+O(\alpha^{2}))(I_{0}+\alpha J+O(\alpha^{2}))=I_{0}+\alpha (J-I_{0}^{2})+O(\alpha^{2}). \end{array}$$ (A.7)

We expand the internal term in (A.3) using (A.7) as:


$$\begin{array}{c} \int_{0}^{\infty }e^{-\alpha I_{\alpha }}I_{\alpha }\;dt=\int_{0}^{\infty }I_{0}\;dt+\alpha \;\left(\int_{0}^{\infty }J\;dt-\int_{0}^{\infty }I_{0}^{2}\;dt\right)+O(\alpha^{2}). \end{array}$$

Plugging in the expansion of $A_{\alpha }$ and $I_{\alpha }(t)$ from (A.5) into (A.1) and comparing α terms gives:


$$\begin{array}{c} \int_{0}^{\infty }J(t)\;dt=\frac{a_{1}}{\gamma +\delta }. \end{array}$$

Hence, the right-hand side of (A.3) can be finally expanded as:


$$\begin{array}{c} -\frac{\beta_{0}}{N}\int_{0}^{\infty }e^{-\alpha I_{\alpha }}I_{\alpha }\;dt=-\frac{\beta_{0}}{N}\frac{A_{0}}{\gamma +\delta }-\frac{\beta_{0}\alpha }{N}\;\left(\frac{a_{1}}{\gamma +\delta }-\int_{0}^{\infty }I_{0}^{2}\;dt\right)+O(\alpha^{2}). \end{array}$$ (A.8)

#### Matching terms.

Now we compare the matching terms of left-hand side expansion, given in (A.6), with right-hand side expansion, given in (A.8). At order *O*(1), we recover the classical relation (A.4).

At order O(α) we have:


$$\begin{array}{c} -\frac{a_{1}}{N-A_{0}}=-\frac{\beta_{0}}{N}\;\left(\frac{a_{1}}{\gamma +\delta }-\int_{0}^{\infty }I_{0}^{2}\;dt\right), \end{array}$$

which can be rewritten as:


$$\begin{array}{c} \left(\frac{1}{N-A_{0}}-\frac{\beta_{0}}{N(\gamma +\delta )}\right)a_{1}=-\frac{\beta_{0}}{N}\int_{0}^{\infty }I_{0}(t{)}^{2}\;dt. \end{array}$$

Therefore, solving for *a*_1_ gives:


$$\begin{array}{c} a_{1}=-\frac{\frac{\beta_{0}}{N}\int_{0}^{\infty }I_{0}(t{)}^{2}\;dt}{\frac{1}{N-A_{0}}-\frac{\beta_{0}}{N(\gamma +\delta )}}. \end{array}$$ (A.9)

Since $A_{\alpha }=A_{0}+\alpha a_{1}+O(\alpha^{2})$, and *A*_0_ corresponds to the final size of the baseline model, to show that the final size of the behavioral model is smaller than that of the baseline model, it is sufficient to show that *a*_1_ < 0. Since we consider small α, the $O(\alpha^{2})$ terms are negligible compared to the first-order term $\alpha a_{1}$, so the sign of *a*_1_ determines whether $A_{\alpha }<A_{0}$ or $A_{\alpha }>A_{0}$.

The numerator of (A.9) is always positive. The denominator of (A.9) is positive provided:


$$\begin{array}{c} N(\gamma +\delta )>\beta_{0}(N-A_{0}), \end{array}$$

which can be rewritten as:


$$\begin{array}{c} {ℛ}_{0}\left(1-\frac{A_{0}}{N}\right)<1, \end{array}$$

which is true since $s_{\infty }=(1-\frac{A_{0}}{N})<\frac{1}{{ℛ}_{0}}$ (see (B.9) for details). This concludes the proof. □

### B Proof of Theorem 3.2

*Proof.* Let $\beta_{\alpha }(t)=\beta_{0}\Psi (\alpha ,I_{\alpha }(t))$, where α=ζδ combines the behavioral sensitivity parameter ζ with the mortality rate δ, and $I_{\alpha }(t)$ denotes the infectious population trajectory corresponding to α. For example, $\Psi (\alpha ,I_{\alpha }(t))=\exp(-\zeta \delta I_{\alpha }(t))=\exp(-\alpha I_{\alpha }(t))$. We require that 0<Ψ(α,I(t))≤1, and that Ψ(0,I(t))=1. We assume that the total population size is fixed with *N*  =  *S* + *E* + *I* + *R* + *D*. Furthermore, initial conditions are: *S*(0) = *S*_0_ > 0, $E(0)=E_{0}\geq 0$, $I(0)=I_{0}\geq 0$, *R*(0) = *D*(0) = 0 and $S_{0}+E_{0}+I_{0}=N$.

In this proof we work with the cumulative number infected, so the *final epidemic size* is defined as


$$\begin{array}{c} A_{\alpha }:=R(\infty )+D(\infty )=(\gamma +\delta )\int_{0}^{\infty }I_{\alpha }(t)\;dt. \end{array}$$ (B.1)

The final-size proportion used in the main text is $A_{\alpha }/N$, so the inequality is unchanged after normalization.

From the *S*–equation of model (2.1), we have


$$\begin{array}{c} \frac{dS}{dt}=-\frac{\beta_{\alpha }(t)}{N}S(t)I_{\alpha }(t). \end{array}$$

Dividing both sides by *S*(*t*) and integrating in time from 0 to ∞ (noting *S*(*t*) > 0 for all finite time until ex*t*inction):


$$\begin{array}{c} \ln\left(\frac{S(\infty )}{S(0)}\right)=-\frac{1}{N}\int_{0}^{\infty }\beta_{\alpha }(t)I_{\alpha }(t)\;dt. \end{array}$$ (B.2)

Since removed individuals at infinity equal the initial population minus susceptibles at infinity, we have


$$\begin{array}{c} S(\infty )=N-A_{\alpha },\;S(0)=S_{0}, \end{array}$$

and hence (B.2) can be rewritten as


$$\begin{array}{c} \ln\left(\frac{N-A_{\alpha }}{S_{0}}\right)=-\frac{1}{N}\int_{0}^{\infty }\beta_{\alpha }(t)I_{\alpha }(t)\;dt. \end{array}$$ (B.3)

Equation (B.3) is exact for any time-dependent Ψ and any solution of the SEIRD model (2.1) satisfying the stated assumptions. Let *A*_0_ denote the final size when Ψ(α=0,I(t))=1.

Recall the pointwise inequality assumption


$$\begin{array}{c} 0<\Psi (\alpha ,I_{\alpha }(t))\leq 1,\;\forall t\geq 0,\;\alpha \geq 0, \end{array}$$

The above implies


$$\begin{array}{c} \beta_{\alpha }(t)=\beta_{0}\Psi (\alpha ,I_{\alpha }(t))\leq \beta_{0}\;\text{for all }t. \end{array}$$

Substituting inequality of $\beta_{\alpha }(t)$ in (B.3) gives:


$$\begin{array}{c} \ln\left(\frac{N-A_{\alpha }}{S_{0}}\right)\geq -\frac{1}{N}\int_{0}^{\infty }\beta_{0}I_{\alpha }(t)\;dt. \end{array}$$ (B.4)

For the baseline case with α=0 (no behavioral response), we have $\beta_{\alpha }(t)=\beta_{0}$ and the infectious trajectory $I_{\alpha }(t)$ reduces to *I*_0_(*t*). Substituting $\beta_{\alpha }(t)=\beta_{0}$ in (B.3) yields:


$$\begin{array}{c} \ln\left(\frac{N-A_{0}}{S_{0}}\right)=-\frac{\beta_{0}}{N}\int_{0}^{\infty }I_{0}(t)\;dt. \end{array}$$ (B.5)

Equations (B.4) and (B.5) cannot be directly compared because they involve both different final epidemic sizes on the left hand side ($A_{\alpha }$ versus *A*_0_) and different infectious population trajectories on the right hand side ($I_{\alpha }(t)$ versus *I*_0_(*t*)). To enable comparison, we apply the relation $\int_{0}^{\infty }I_{\alpha }(t)\;dt=A_{\alpha }/(\gamma +\delta )$ from equation (B.1), which transforms inequality (B.4) into:


$$\begin{array}{c} \ln\left(\frac{N-A_{\alpha }}{S_{0}}\right)\geq -\frac{\beta_{0}}{N}\cdot \frac{A_{\alpha }}{\gamma +\delta }, \end{array}$$ (B.6)

and for baseline case, equation (B.5) can rewritten as:


$$\begin{array}{c} \ln\left(\frac{N-A_{0}}{S_{0}}\right)=-\frac{\beta_{0}}{N}\cdot \frac{A_{0}}{\gamma +\delta }. \end{array}$$ (B.7)

We define the function


$$\begin{array}{c} f(A):=\ln\left(\frac{N-A}{S_{0}}\right)+\frac{\beta_{0}}{N(\gamma +\delta )}A. \end{array}$$ (B.8)

Notice that for the baseline model *f*(*A*_0_) = 0 while for behavioral models $f(A_{\alpha })\geq 0$. *f*(*A*) has two zeros, the trivial solution *A* = 0 (no epidemic) and the nontrivial solution *A* = *A*_0_ (baseline epidemic). The proof proceeds in three steps. First, we show *f*(*A*) is strictly concave and compute its unique maximum at $A^{*}=N(1-1/{ℛ}_{0})$. Second, since *A*_0_ is defined only implicitly through a transcendental equation (B.7), we cannot directly compare *A*_0_ with $A^{*}$. We therefore normalize the susceptible population as *s* = *S*/*N* and express the baseline final size through the transcendental equation $\ln s_{\infty }=-{ℛ}_{0}(1-s_{\infty })$, where $s_{\infty }=(N-A_{0})/N$. By analyzing the auxiliary function $\Phi (s):=\ln s+{ℛ}_{0}(1-s)$, we show that it attains a unique maximum at $s=1/{ℛ}_{0}$. Furthermore, we prove that $s_{\infty }<1/{ℛ}_{0}$, which translates to $A_{0}>A^{*}$. Third, combining $A_{0}>A^{*}$ with the concavity of *f*(*A*) and the inequality $f(A_{\alpha })\geq 0$, we conclude $A_{\alpha }\leq A_{0}$. Details follow.

Differentiating *f*(*A*) with respect to *A* gives (where $c=\frac{\beta_{0}}{N(\gamma +\delta )}$ is a constant):


$$\begin{array}{c} f^{\prime }(A)=-\frac{1}{N-A}+c,\;f^{\prime \prime }(A)=-\frac{1}{(N-A{)}^{2}}<0\;\text{for }0\leq A<N. \end{array}$$

Thus *f* is strictly concave on [0,*N*) and can have at most one critical point (which will be a global maximum).

Solving $f^{\prime }(A)=0$ yields $-\frac{1}{N-A}+c=0$, which gives $N-A=\frac{1}{c}$. Thus the unique critical point is


$$\begin{array}{c} A^{*}=N-\frac{1}{c}, \end{array}$$

where $c={ℛ}_{0}/N$. Because $f^{\prime \prime }(A)<0$, $A^{*}$ is the global maximizer of *f* on [0,*N*).

#### Relation to *A*_0_.

Introduce the normalized susceptible fraction *s*: = *S*/*N*. We use the standard approximation $S_{0}\approx N$, consistent with the assumption of a nearly fully susceptible population at epidemic onset. It follows from (B.7) that the classical final-size equation for α=0 can be written in normalized form as


$$\begin{array}{c} \ln s_{\infty }=-{ℛ}_{0}(1-s_{\infty }),\;\text{with}\;s_{\infty }:=\frac{S(\infty )}{N}=\frac{N-A_{0}}{N}. \end{array}$$

Define the auxiliary function


$$\begin{array}{c} \Phi (s):=\ln s+{ℛ}_{0}(1-s),\;s\in (0,1]. \end{array}$$

Then $s_{\infty }$ is a zero of Φ, i.e., $\Phi (s_{\infty })=0$.

Compute the derivative:


$$\begin{array}{c} \Phi^{\prime }(s)=\frac{1}{s}-{ℛ}_{0}. \end{array}$$

Hence $\Phi^{\prime }(s)>0$ for $0<s<1/{ℛ}_{0}$, $\Phi^{\prime }(1/{ℛ}_{0})=0$, and $\Phi^{\prime }(s)<0$ for $s>1/{ℛ}_{0}$. Thus Φ attains its unique maximum at $s=1/{ℛ}_{0}$.

Evaluate Φ at the endpoints and at the maximum:


$$\begin{array}{c} \Phi (1)=\ln1+{ℛ}_{0}(1-1)=0, \end{array}$$

and by the standard inequality lnx≤x−1 (with equality only at *x* = 1),


$$\begin{array}{c} \Phi \;(1/{ℛ}_{0})=\ln\;(1/{ℛ}_{0})+{ℛ}_{0}\;(1-\frac{1}{{ℛ}_{0}})=-\ln{ℛ}_{0}+{ℛ}_{0}-1\geq 0, \end{array}$$

with strict inequality when ${ℛ}_{0}\neq 1$. Thus the maximum value $\Phi (1/{ℛ}_{0})$ is nonnegative (positive for ${ℛ}_{0}>1$) while Φ(1)=0.

Because Φ is continuous on (0,1], has a unique maximum at $1/{ℛ}_{0}$ with $\Phi (1/{ℛ}_{0})\geq 0$, and Φ(1)=0, the equation Φ(s)=0 has exactly two solutions in (0,1] when $\Phi (1/{ℛ}_{0})>0$: one at *s* = 1 and the other strictly to the left of $1/{ℛ}_{0}$. The epidemiologically relevant solution is the nontrivial root $s_{\infty }\in (0,1)$. Because $\Phi (1/{ℛ}_{0})>0$ and Φ(1)=0, this nontrivial root must satisfy


$$\begin{array}{c} s_{\infty }<\frac{1}{{ℛ}_{0}}. \end{array}$$ (B.9)

Conclude $A^{*}<A_{0}$. Translate back to *A*. Since $s_{\infty }=(N-A_{0})/N$, the inequality $s_{\infty }<1/{ℛ}_{0}$ is equivalent to


$$\begin{array}{c} \frac{N-A_{0}}{N}<\frac{1}{{ℛ}_{0}}\;⟹\;N-A_{0}<\frac{N}{{ℛ}_{0}}=\frac{1}{c}, \end{array}$$

hence


$$\begin{array}{c} A_{0}>N-\frac{1}{c}=A^{*}. \end{array}$$

Therefore the unique maximizer $A^{*}$ of *f*(*A*), given by (B.8), indeed lies strictly to the left of the classical final size *A*_0_ (for ${ℛ}_{0}>1$, i.e., in the nontrivial epidemic regime). Since $A_{\alpha }$ satisfies inequality (B.6), we conclude that $A_{\alpha }$ must lie to the left of *A*_0_. Hence, $A_{\alpha }\leq A_{0}$. □

## Supporting information

S1 Appendix Supplementary methods and results. (PDF)
