# Supplementary material for: The paradox of neglecting changes in behavior: How standard epidemic models misestimate both transmissibility and final epidemic size
Source: PLoS Comput Biol. 2026 Sep 3;22(9):e1014746. doi: 10.1371/journal.pcbi.1014746 (PMC13568531; doi:10.1371/journal.pcbi.1014746)
Supplement: Supplementary file 1 [file pcbi.1014746.s001.pdf]

Supplementary Materials for
"The Paradox of Neglecting Changes in Behavior: How Standard Epidemic Models Misestimate Both Transmissibility and Final Epidemic Size"

Binod Pant[1,2,3], Marko Lalovic[4], István Z. Kiss[4,5], and Mauricio Santillana[2,3,6*]

[1] Department of Mathematics and Statistics, Mississippi State University, Mississippi State, Mississippi, United States of America

[2] Machine Intelligence Group for the Betterment of Health and the Environment, Northeastern University, Boston, Massachusetts, United States of America

[3] Network Science Institute, Northeastern University, Boston, Massachusetts, United States of America

[4] Network Science Institute, Northeastern University London, London, United Kingdom

[5] Department of Mathematics, Northeastern University, Boston, Massachusetts, United States of America

[6] Department of Epidemiology, Harvard T.H. Chan School of Public Health, Boston, Massachusetts, United States of America

These authors contributed equally to this work.

* msantill@g.harvard.edu

Contents

A1 Location Abbreviations **3**

A2 Excluded U.S. Locations and Sensitivity to Location-Selection Criteria **4**

A3 Parameter Inference Details **9**

A4 Synthetic Data Experiment Details **10**

A5 Exponential and Rational Behavioral Models **13**

A6 Effective Transmission Rate **19**

A7 Effective Reproduction Number **22**

List of Figures

A1 COVID-19 mortality in excluded US locations . . . 4
A2 Synthetic experiment for behavioral (Exponential) model . . . 11
A3 Synthetic experiment for behavioral (Rational) model . . . 12
A4 Posterior predictive fits for behavioral (Exponential) model . . . 13
A5 Posterior predictive fits for behavioral (Rational) model . . . 14
A6 Basic reproduction number and final size for behavioral (Exponential) model . . . 15
A7 Basic reproduction number and final size for behavioral (Rational) model . . . 16
A8 Effective transmission rates: Mixed model . . . 19
A9 Effective transmission rates: Exponential model . . . 20
A10 Effective transmission rates: Rational model . . . 21
A11 Effective reproduction number: Mixed model . . . 22
A12 Effective reproduction number: Exponential model . . . 23
A13 Effective reproduction number: Rational model . . . 24

List of Tables

A1 Full names and standard two-letter abbreviations for all 51 US locations considered in the analysis. . . . 3
A2 Locations excluded from the analysis. . . . 5
A3 Mixed-model sensitivity analysis across all 51 locations. . . . 6
A4 Exponential-model sensitivity analysis across all 51 locations. . . . 7
A5 Rational-model sensitivity analysis across all 51 locations. . . . 8
A6 Comparison of the baseline model and the behavioral (Exponential) model across 20 US locations. . . . 17
A7 Comparison of the baseline model and the behavioral (Rational) model across 20 US locations. 18

A1 Location Abbreviations

Table A1. Full names and standard two-letter abbreviations for all 51 US locations considered in the analysis.

Location	Abbreviation	Location	Abbreviation
Alaska	AK	Nebraska	NE
Alabama	AL	Nevada	NV
Arkansas	AR	New Hampshire	NH
Arizona	AZ	New Jersey	NJ
California	CA	New Mexico	NM
Colorado	CO	New York	NY
Connecticut	CT	North Carolina	NC
Delaware	DE	North Dakota	ND
District of Columbia	DC	Ohio	OH
Florida	FL	Oklahoma	OK
Georgia	GA	Oregon	OR
Hawaii	HI	Pennsylvania	PA
Idaho	ID	Rhode Island	RI
Illinois	IL	South Carolina	SC
Indiana	IN	South Dakota	SD
Iowa	IA	Tennessee	TN
Kansas	KS	Texas	TX
Kentucky	KY	Utah	UT
Louisiana	LA	Vermont	VT
Maine	ME	Virginia	VA
Maryland	MD	Washington	WA
Massachusetts	MA	West Virginia	WV
Michigan	MI	Wisconsin	WI
Minnesota	MN	Wyoming	WY
Mississippi	MS	Missouri	MO
Montana	MT		

A2 Excluded U.S. Locations and Sensitivity to Location-Selection Criteria

The primary analysis focused on 20 locations whose first-wave mortality trajectories carried sufficient signal and showed a sufficiently resolved rise-and-decline pattern for single-wave inference. The excluded locations are shown here for transparency. We report the primary screening obstacle for each excluded location and provide sensitivity analyses comparing model-selection, $\mathcal{R}_0$, and final-size estimates across all 51 locations. These additional results are intended to assess robustness to the location-selection procedure. Estimates from excluded locations should be interpreted cautiously because their mortality trajectories fail one or more screening criteria.

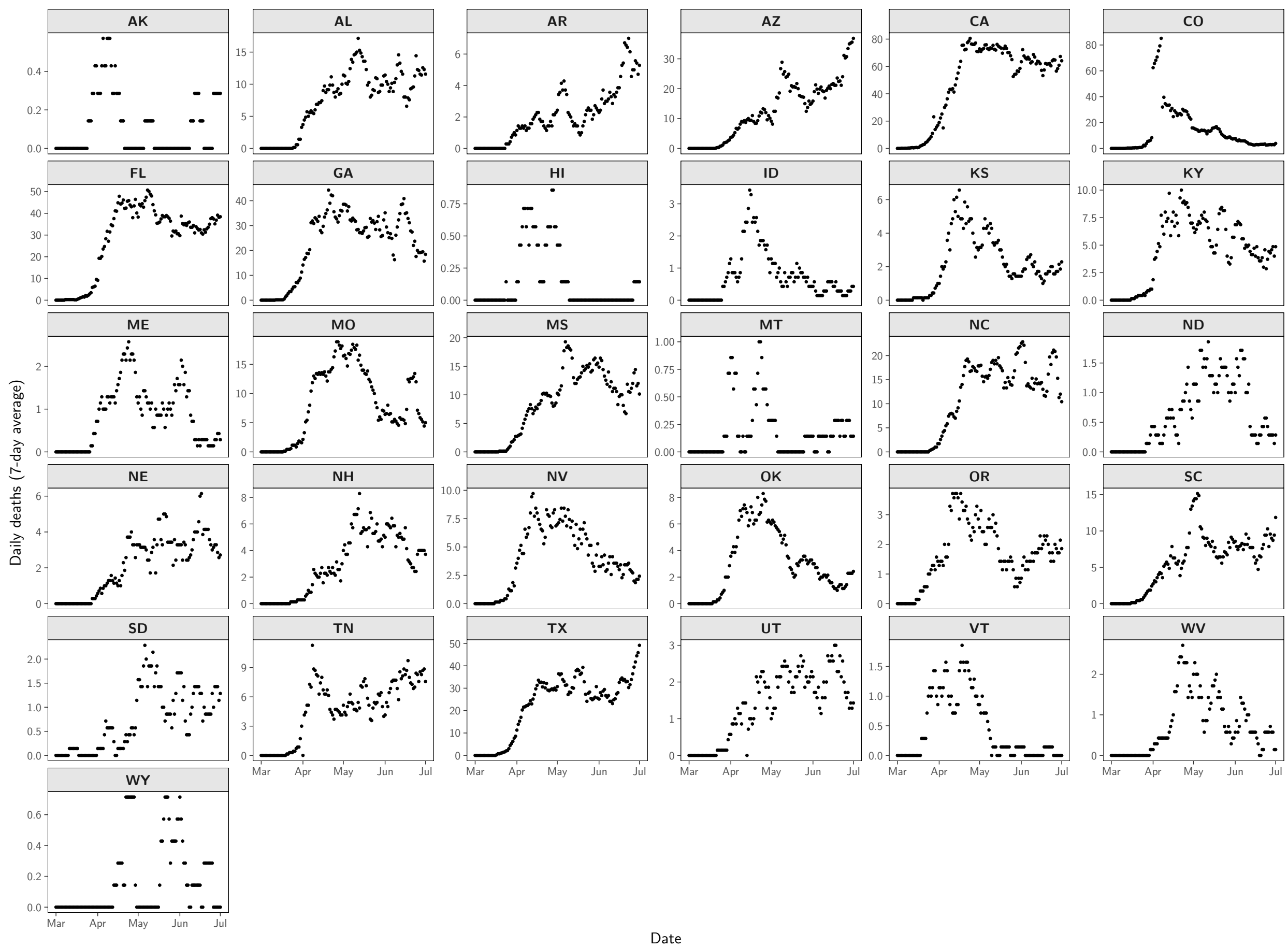

Fig A1. COVID-19 mortality time series for US locations excluded from the main analysis. Panels show observed 7-day averaged daily COVID-19 deaths for the 31 excluded US locations, ordered alphabetically. Locations were excluded because of low mortality signal, absence of a completed first mortality wave within the observation window, or a reporting anomaly. See Methods: Data and Location Selection 2.2 and Table A2.

Table A2. Locations excluded from the primary analysis and the primary data-fitting obstacle identified by the pre-specified mortality-trajectory screening criteria. Here $y(t)$ denotes the 7-day moving average of daily deaths over the March 1–July 1, 2020 analysis window. A location was excluded for low signal if $\max_t y(t) \leq 10$ or $\sum_t y(t) < 50$; for an incomplete mortality wave if the mortality peak occurred fewer than 21 days before the end of the observation window or if the mean mortality over the final 14 days exceeded 40% of the peak. Colorado was excluded because of a reporting discontinuity in cumulative mortality data producing nonphysical daily increments after differencing. "Tail/peak" is the ratio of the final-14-day mean to the peak.

Location	Primary reason	$\max_t y(t)$	Tail/peak	Diagnostic note
AK	Low signal	0.57	0.23	peak ≤ 10
AR	Low signal	7.00	0.81	peak ≤ 10
HI	Low signal	0.86	0.06	peak ≤ 10
ID	Low signal	3.43	0.08	peak ≤ 10
KS	Low signal	6.57	0.27	peak ≤ 10
KY	Low signal	10.00	0.40	peak ≤ 10
ME	Low signal	2.57	0.10	peak ≤ 10
MT	Low signal	1.00	0.22	peak ≤ 10
ND	Low signal	1.86	0.18	peak ≤ 10
NE	Low signal	6.14	0.56	peak ≤ 10
NH	Low signal	8.29	0.41	peak ≤ 10
NV	Low signal	9.71	0.28	peak ≤ 10
OK	Low signal	8.29	0.20	peak ≤ 10
OR	Low signal	3.71	0.51	peak ≤ 10
SD	Low signal	2.29	0.47	peak ≤ 10
UT	Low signal	3.00	0.65	peak ≤ 10
VT	Low signal	1.86	0.04	peak ≤ 10
WV	Low signal	2.71	0.13	peak ≤ 10
WY	Low signal	0.71	0.20	peak ≤ 10
AL	Incomplete wave	17.14	0.64	tail/peak > 0.4
AZ	Incomplete wave	36.86	0.78	peak too late
CA	Incomplete wave	80.57	0.77	tail/peak > 0.4
FL	Incomplete wave	50.71	0.70	tail/peak > 0.4
GA	Incomplete wave	44.29	0.51	tail/peak > 0.4
MO	Incomplete wave	18.86	0.48	tail/peak > 0.4
MS	Incomplete wave	19.29	0.56	tail/peak > 0.4
NC	Incomplete wave	22.86	0.69	tail/peak > 0.4
SC	Incomplete wave	15.14	0.56	tail/peak > 0.4
TN	Incomplete wave	11.29	0.71	tail/peak > 0.4
TX	Incomplete wave	49.14	0.76	peak too late
CO	Reporting anomaly	85.14	0.04	reporting discontinuity

Table A3. Sensitivity analysis comparing the baseline model and the behavioral mixed model across all 51 US locations. The 20 retained locations form the primary analysis set. The 31 excluded locations are shown only to assess robustness to the location-selection procedure. Estimates for excluded locations should be interpreted with caution because their mortality trajectories fail one or more data-screening criteria described in Table A2. Median $NSSE_B$ and Median $NSSE_M$ denote the median posterior normalized sum of squared errors under the baseline and behavioral mixed models, respectively. The columns $\mathcal{R}_{0,M} - \mathcal{R}_{0,B}$ and $A_B - A_M$ report differences in posterior medians. Positive values indicate that the baseline model estimates a lower basic reproduction number and a higher final epidemic size than the behavioral mixed model. Negative values may occur in excluded locations whose mortality trajectories violate the assumptions of coherent single-wave structure. BF_{eq} is the Bayes factor under equal model priors in favor of the mixed model versus the baseline model. Table is ordered by decreasing Bayes factor in favor of the behavioral model. Bold values indicate the lower median NSSE within each row.

Location	Set	Median $NSSE_B$	Median $NSSE_M$	$\mathcal{R}_{0,M} - \mathcal{R}_{0,B}$	$A_B - A_M$	BF_{eq}	Evidence
DC	Included	0.0286	**0.0133**	1.45	0.018	11551.24	very strong
RI	Included	0.0251	**0.0107**	0.69	0.035	852.43	very strong
NM	Included	0.0493	**0.0151**	0.80	0.061	688.87	very strong
IN	Included	0.0900	**0.0087**	1.25	0.068	126.43	strong
CT	Included	0.0331	**0.0121**	2.41	0.006	121.80	strong
WI	Included	0.2540	**0.0559**	1.26	0.079	105.04	strong
VA	Included	0.0815	**0.0388**	1.37	0.048	100.58	strong
MN	Included	0.0398	**0.0185**	0.79	0.052	79.31	strong
WA	Included	0.1784	**0.0656**	1.19	0.075	64.52	strong
OH	Included	0.0685	**0.0058**	1.03	0.066	37.48	strong
IL	Included	0.0574	**0.0118**	0.94	0.066	35.61	strong
MA	Included	0.0377	**0.0143**	2.44	0.006	22.96	strong
PA	Included	0.0472	**0.0311**	1.66	0.017	19.86	positive
LA	Included	0.0837	**0.0166**	2.11	0.045	13.46	positive
MD	Included	0.0617	**0.0152**	2.67	0.009	11.97	positive
MI	Included	0.0345	**0.0123**	1.94	0.023	11.80	positive
DE	Included	0.0214	**0.0167**	0.55	0.018	10.67	positive
NJ	Included	0.0430	**0.0149**	2.05	0.024	3.67	positive
IA	Included	**0.0139**	0.0168	0.15	0.012	0.51	favors Baseline
NY	Included	**0.0277**	0.0310	1.00	0.012	0.02	favors Baseline
MS	Excluded	0.0874	**0.0338**	0.43	0.137	5051.63	very strong
AL	Excluded	0.1524	**0.0372**	1.99	-0.394	3106.31	very strong
NH	Excluded	0.0569	**0.0378**	0.28	0.083	2105.67	very strong
NE	Excluded	0.1405	**0.0863**	0.11	0.108	1852.99	very strong
TN	Excluded	0.1408	**0.1049**	0.73	0.269	1617.90	very strong
TX	Excluded	0.1274	**0.0255**	4.45	0.266	1568.66	very strong
SC	Excluded	0.1881	**0.0703**	1.63	-0.380	955.14	very strong
MO	Excluded	0.1207	**0.0539**	2.49	0.037	211.95	very strong
KY	Excluded	0.2150	**0.0581**	0.58	0.107	210.55	very strong
NC	Excluded	0.1444	**0.0310**	2.69	0.027	164.63	very strong
FL	Excluded	0.1947	**0.0116**	4.51	-0.522	110.03	strong
CA	Excluded	0.1699	**0.0074**	2.80	0.028	98.43	strong
UT	Excluded	0.1423	**0.1088**	0.08	0.057	85.85	strong
AZ	Excluded	0.0786	**0.0639**	0.65	0.223	79.86	strong
GA	Excluded	0.2248	**0.0266**	3.23	0.024	66.31	strong
OR	Excluded	0.2407	**0.1922**	0.10	0.037	57.95	strong
OK	Excluded	0.1005	**0.0387**	0.48	0.065	28.71	strong
ME	Excluded	0.2243	**0.2012**	0.03	0.022	23.49	strong
VT	Excluded	0.1672	**0.1584**	0.14	0.004	6.00	positive
AR	Excluded	**0.0888**	0.0901	0.04	0.140	2.23	very weak
NV	Excluded	0.1455	**0.0352**	0.61	0.088	1.47	very weak
ND	Excluded	0.0938	**0.0904**	0.04	0.017	1.43	very weak
KS	Excluded	0.1629	**0.1063**	0.21	0.053	1.34	very weak
SD	Excluded	0.1907	**0.1731**	0.04	0.029	1.08	very weak
WV	Excluded	0.1515	**0.1402**	0.10	0.018	1.06	very weak
HI	Excluded	0.2382	**0.2373**	0.12	0.000	1.00	very weak
WY	Excluded	**0.5176**	0.5179	0.00	0.004	1.00	favors Baseline
MT	Excluded	0.5139	**0.5137**	0.02	0.001	0.98	favors Baseline
ID	Excluded	0.1068	**0.1060**	0.31	0.003	0.98	favors Baseline
AK	Excluded	**0.3156**	0.3167	-0.01	0.000	0.96	favors Baseline
CO	Excluded	**0.2157**	0.2182	-0.09	0.015	0.24	favors Baseline

Table A4. Sensitivity analysis comparing the baseline model and the behavioral exponential model across all 51 US locations. The 20 retained locations form the primary analysis set. The 31 excluded locations are shown only to assess robustness to the location-selection procedure. Estimates for excluded locations should be interpreted with caution because their mortality trajectories fail one or more data-screening criteria described in Table A2. Median $\mathrm{NSSE_B}$ and Median $\mathrm{NSSE_M}$ denote the median posterior normalized sum of squared errors under the baseline and behavioral exponential models, respectively. The columns $\mathcal{R}_{0,M} - \mathcal{R}_{0,B}$ and $A_B - A_M$ report differences in posterior medians. Positive values indicate that the baseline model estimates a lower basic reproduction number and a higher final epidemic size than the behavioral exponential model. $\mathrm{BF_{eq}}$ is the Bayes factor under equal model priors in favor of the exponential model versus the baseline model. Table is ordered by decreasing Bayes factor in favor of the behavioral model. Bold values indicate the lower median NSSE within each row.

Location	Set	Median $\mathrm{NSSE_B}$	Median $\mathrm{NSSE_M}$	$\mathcal{R}_{0,M} - \mathcal{R}_{0,B}$	$A_B - A_M$	$\mathrm{BF_{eq}}$	Evidence
VA	Included	0.0815	**0.0385**	1.21	0.049	9343.72	very strong
RI	Included	0.0251	**0.0107**	0.65	0.036	529.25	very strong
WI	Included	0.2540	**0.1144**	0.64	0.063	335.87	very strong
OH	Included	0.0685	**0.0053**	0.86	0.070	190.33	very strong
NM	Included	0.0493	**0.0189**	0.20	0.072	124.12	strong
IN	Included	0.0900	**0.0086**	1.02	0.074	104.37	strong
CT	Included	0.0331	**0.0106**	2.49	0.005	69.45	strong
IL	Included	0.0574	**0.0111**	0.78	0.072	61.22	strong
MN	Included	0.0398	**0.0184**	0.75	0.051	59.66	strong
MD	Included	0.0617	**0.0146**	2.65	0.004	26.79	strong
PA	Included	0.0472	**0.0311**	1.53	0.018	20.64	strong
NJ	Included	0.0430	**0.0125**	2.11	0.021	18.37	positive
MA	Included	0.0377	**0.0133**	2.47	0.003	16.73	positive
DE	Included	0.0214	**0.0164**	0.47	0.019	16.53	positive
LA	Included	0.0837	**0.0160**	2.12	0.043	16.33	positive
WA	Included	0.1784	**0.0675**	0.83	0.071	8.05	positive
MI	Included	0.0345	**0.0112**	2.02	0.021	8.05	positive
DC	Included	0.0286	**0.0144**	0.64	0.030	1.17	very weak
IA	Included	**0.0139**	0.0161	0.11	0.010	0.79	favors Baseline
NY	Included	0.0277	**0.0263**	1.07	0.010	0.03	favors Baseline
NH	Excluded	0.0569	**0.0405**	0.10	0.060	20035.31	very strong
TN	Excluded	0.1408	**0.1151**	0.31	0.158	4672.98	very strong
NC	Excluded	0.1444	**0.0362**	0.84	0.115	3660.24	very strong
CA	Excluded	0.1699	**0.0075**	2.54	0.037	2721.70	very strong
MO	Excluded	0.1207	**0.0574**	0.84	0.077	2326.63	very strong
UT	Excluded	0.1423	**0.1264**	0.03	0.031	699.88	very strong
GA	Excluded	0.2248	**0.0274**	2.94	0.028	577.91	very strong
TX	Excluded	0.1274	**0.0260**	3.49	0.234	380.08	very strong
MS	Excluded	0.0874	**0.0337**	0.32	0.140	133.93	strong
FL	Excluded	0.1947	**0.0110**	4.54	-0.526	69.67	strong
AL	Excluded	0.1524	**0.0477**	1.30	-0.370	60.34	strong
SC	Excluded	0.1881	**0.0975**	1.35	-0.426	48.64	strong
AZ	Excluded	0.0786	**0.0642**	0.61	0.235	39.83	strong
KY	Excluded	0.2150	**0.1118**	0.26	0.068	31.04	strong
AR	Excluded	**0.0888**	0.0894	0.02	0.126	1.58	very weak
NE	Excluded	0.1405	**0.1108**	0.06	0.050	1.27	very weak
NV	Excluded	0.1455	**0.0745**	0.28	0.052	1.17	very weak
OK	Excluded	0.1005	**0.0625**	0.22	0.034	1.17	very weak
VT	Excluded	0.1672	**0.1631**	0.06	0.002	1.07	very weak
KS	Excluded	0.1629	**0.1334**	0.11	0.025	1.06	very weak
OR	Excluded	0.2407	**0.2190**	0.06	0.016	1.06	very weak
ME	Excluded	0.2243	**0.2242**	0.09	0.003	1.06	very weak
SD	Excluded	0.1907	**0.1816**	0.01	0.013	1.05	very weak
ID	Excluded	0.1068	**0.1064**	0.14	0.002	1.04	very weak
WY	Excluded	**0.5176**	0.5176	0.00	0.002	1.03	very weak
ND	Excluded	0.0938	**0.0916**	0.01	0.009	1.02	very weak
WV	Excluded	0.1515	**0.1462**	0.05	0.008	1.01	very weak
HI	Excluded	0.2382	**0.2378**	0.06	0.000	0.99	favors Baseline
MT	Excluded	0.5139	**0.5138**	0.01	0.001	0.98	favors Baseline
AK	Excluded	**0.3156**	0.3160	-0.00	0.000	0.97	favors Baseline
CO	Excluded	**0.2157**	0.2163	-0.04	0.019	0.55	favors Baseline

Table A5. Sensitivity analysis comparing the baseline model and the behavioral rational model across all 51 US locations. The 20 retained locations form the primary analysis set. The 31 excluded locations are shown only to assess robustness to the location-selection procedure. Estimates for excluded locations should be interpreted with caution because their mortality trajectories fail one or more data-screening criteria described in Table A2. Median $NSSE_B$ and Median $NSSE_M$ denote the median posterior normalized sum of squared errors under the baseline and behavioral rational models, respectively. The columns $\mathcal{R}_{0,M} - \mathcal{R}_{0,B}$ and $A_B - A_M$ report differences in posterior medians. Positive values indicate that the baseline model estimates a lower basic reproduction number and a higher final epidemic size than the behavioral rational model. BF_{eq} is the Bayes factor under equal model priors in favor of the rational model versus the baseline model. Table is ordered by decreasing Bayes factor in favor of the behavioral model. Bold values indicate the lower median NSSE within each row.

Location	Set	Median $NSSE_B$	Median $NSSE_M$	$\mathcal{R}_{0,M} - \mathcal{R}_{0,B}$	$A_B - A_M$	BF_{eq}	Evidence
MN	Included	0.0398	**0.0178**	0.90	0.053	766.21	very strong
MI	Included	0.0345	**0.0112**	2.18	0.023	618.29	very strong
RI	Included	0.0251	**0.0098**	0.77	0.036	580.79	very strong
VA	Included	0.0815	**0.0384**	1.22	0.059	551.33	very strong
LA	Included	0.0837	**0.0168**	2.30	0.052	484.47	very strong
MD	Included	0.0617	**0.0153**	3.00	0.016	260.53	very strong
WI	Included	0.2540	**0.1431**	0.46	0.056	235.46	very strong
IL	Included	0.0574	**0.0087**	1.13	0.069	147.28	strong
IN	Included	0.0900	**0.0070**	1.74	0.067	99.39	strong
OH	Included	0.0685	**0.0045**	1.44	0.063	75.25	strong
PA	Included	0.0472	**0.0292**	2.33	0.014	49.68	strong
MA	Included	0.0377	**0.0138**	2.68	0.010	23.95	strong
NJ	Included	0.0430	**0.0124**	2.33	0.026	22.78	strong
NM	Included	0.0493	**0.0219**	0.16	0.063	21.02	strong
WA	Included	0.1784	**0.1001**	0.49	0.053	20.84	strong
DE	Included	0.0214	**0.0162**	0.45	0.020	15.75	positive
IA	Included	**0.0139**	0.0159	0.12	0.011	2.37	very weak
CT	Included	0.0331	**0.0122**	2.57	0.010	1.76	very weak
DC	Included	0.0286	**0.0159**	0.46	0.032	1.15	very weak
NY	Included	0.0277	**0.0265**	1.03	0.010	0.04	favors Baseline
GA	Excluded	0.2248	**0.0361**	1.24	0.111	21622.14	very strong
NE	Excluded	0.1405	**0.1153**	0.06	0.044	684.73	very strong
UT	Excluded	0.1423	**0.1279**	0.03	0.027	607.78	very strong
MO	Excluded	0.1207	**0.0634**	0.45	0.084	378.67	very strong
CA	Excluded	0.1699	**0.0095**	3.15	0.030	323.43	very strong
SC	Excluded	0.1881	**0.1188**	1.32	-0.446	196.83	very strong
TX	Excluded	0.1274	**0.0400**	1.82	-0.364	181.03	very strong
MS	Excluded	0.0874	**0.0348**	0.26	0.128	114.97	strong
NC	Excluded	0.1444	**0.0447**	0.39	0.147	113.90	strong
TN	Excluded	0.1408	**0.1173**	0.26	0.143	86.81	strong
AL	Excluded	0.1524	**0.0641**	1.29	-0.406	85.14	strong
FL	Excluded	0.1947	**0.0209**	2.96	-0.460	83.48	strong
KY	Excluded	0.2150	**0.1307**	0.21	0.057	48.24	strong
AZ	Excluded	0.0786	**0.0620**	0.81	0.252	46.76	strong
NV	Excluded	0.1455	**0.0865**	0.23	0.043	38.68	strong
OK	Excluded	0.1005	**0.0679**	0.19	0.029	36.42	strong
KS	Excluded	0.1629	**0.1362**	0.08	0.022	12.89	positive
WV	Excluded	0.1515	**0.1462**	0.04	0.008	3.59	positive
NH	Excluded	0.0569	**0.0418**	0.09	0.053	1.24	very weak
VT	Excluded	0.1672	**0.1629**	0.05	0.002	1.22	very weak
CO	Excluded	**0.2157**	0.2184	-0.06	0.005	1.14	very weak
SD	Excluded	0.1907	**0.1821**	0.02	0.012	1.07	very weak
OR	Excluded	0.2407	**0.2218**	0.07	0.014	1.06	very weak
WY	Excluded	0.5176	**0.5175**	0.00	0.003	1.04	very weak
ND	Excluded	0.0938	**0.0920**	0.02	0.008	1.03	very weak
AK	Excluded	**0.3156**	0.3163	-0.01	0.000	1.01	very weak
ID	Excluded	0.1068	**0.1065**	0.10	0.001	1.00	very weak
HI	Excluded	0.2382	**0.2378**	0.05	-0.000	0.99	favors Baseline
ME	Excluded	0.2243	**0.2149**	0.03	0.009	0.99	favors Baseline
MT	Excluded	0.5139	**0.5137**	0.00	0.001	0.96	favors Baseline
AR	Excluded	**0.0888**	0.0896	0.03	0.141	0.39	favors Baseline

A3 Parameter Inference Details

This section provides additional implementation details that complement the parameter inference description in the main text (Section 2.3), without repeating the full specification of the model, priors, or ABC–SMC setup.

Data and preprocessing. For each location, we work with location-level COVID-19 mortality over the period of $T = 123$ days from March 1 to July 1, 2020. Let $D_{\mathrm{obs}}(t)$ denote cumulative reported deaths. Observed daily mortality counts are obtained as

$$M_{\mathrm{obs}}(t) = D_{\mathrm{obs}}(t) - D_{\mathrm{obs}}(t-1). \tag{A3.1}$$

To suppress reporting artifacts such as weekend backlogs, we apply a trailing 7-day moving average,

$$\widetilde{M}(t) = \frac{1}{7}\sum_{k=0}^{6} M(t-k). \tag{A3.2}$$

Because this average uses the current day and the previous six days, smoothing is computed using a six-day padding interval before March 1, 2020. The padded values are used only to define the moving average at the beginning of the analysis window and are not included in the fitted time series. The final observed series therefore covers exactly the target interval from March 1 to July 1, 2020.

The same preprocessing is applied to model-simulated trajectories before computing distances. Simulated daily deaths are computed from the SEIRD system (Eq. (2.1)), smoothed using the same trailing 7-day moving average (A3.2), and aligned to the same T-day fitting window as the data. Thus, the endpoint of the analysis remains fixed at July 1, 2020 and the padding is only a preprocessing device needed to apply the moving average consistently at the boundary.

Fixed parameters. Fixed parameters are chosen based on early SARS-CoV-2 data. The incubation rate was set to $\sigma = 1/3$ day^{-1} (average latent period of 3 days) [1, 2], and the recovery rate to $\gamma = 1/10$ day^{-1} (average infectious period of 10 days) [3, 4]. The disease-induced mortality rate δ is inferred rather than fixed.

The total population size N is location-specific and obtained from the processed Johns Hopkins CSSE data. Initial conditions are fixed at $E_0 = 1$, $R_0 = 0$, and $D_0 = 0$, with I_0 determined from the inferred initial prevalence and $S_0 = N - (E_0 + I_0 + R_0)$.

Inferred parameters and reparameterization. As described in the main text, we reparameterize the model in terms of the basic reproduction number $\mathcal{R}_0$ and the initial prevalence $\pi_0 = I_0/N$ rather than directly estimating β_0 and I_0. At each evaluation,

$$\beta_0 = \mathcal{R}_0(\gamma + \delta), \qquad I_0 = \max\{1,\, N\pi_0\},$$

so that $\mathcal{R}_0$ retains its standard interpretation as the basic reproduction number in a fully susceptible population (Eq. (2.6)).

The priors on $\mathcal{R}_0, \pi_0, \delta, \zeta$ are given in Section 2.3. Here we simply note that log-uniform (Jeffreys-type) priors on π_0 and δ are used to allow these positive parameters to vary over several orders of magnitude without favoring any particular scale a priori, which is appropriate when both are expected to be small and poorly constrained before observing the data.

Prior choice for the behavioral response parameter. The behavioral parameter ζ is not directly observable and should not be interpreted as an empirical estimate of a specific behavioral process. Instead, it is a phenomenological parameter controlling the strength of the feedback from disease-induced mortality to transmission reduction. In all three behavioral formulations, the feedback depends on the dimensionless quantity

$$x(t) = \zeta\delta I(t),$$

where $\delta I(t)$ is the instantaneous mortality rate. The prior $\zeta \sim \mathcal{U}(0, 0.05)$ was chosen by prior-predictive scale considerations. At $\zeta = 0$, the behavioral models reduce exactly to the baseline model. At the upper bound, the model permits strong transmission reduction at mortality levels comparable to those observed during the first pandemic wave. Specifically, for mortality rates $m = \delta I(t)$ of 10, 50, and 100 deaths per day, the largest prior value gives $x = 0.5, 2.5$, and 5, respectively. These values correspond to exponential transmission multipliers e^{-x} of approximately 0.61, 0.08, and 0.007; rational multipliers $(1+x)^{-1}$ of approximately 0.67, 0.29, and 0.17; and mixed multipliers $e^{-x}(1+x)^{-1}$ of approximately 0.40, 0.02, and 0.001. Thus the prior allows responses ranging from negligible to strong while preserving nonnegative transmission rates.

Because ζ is scale-dependent, posterior estimates of ζ should be interpreted conditionally on the chosen mortality-driven feedback form rather than as direct measurements of behavioral sensitivity.

Parameter estimation via ABC–SMC. We compute the discrepancy between simulated $\boldsymbol{y}_{\text{sim}}(\boldsymbol{\theta}) = \left(\widetilde{M}_{\text{sim}}(t; \boldsymbol{\theta})\right)_{t=1}^{T}$ and observed smoothed mortality series $\boldsymbol{y}_{\text{obs}} = \left(\widetilde{M}_{\text{obs}}(t)\right)_{t=1}^{T}$ using the normalized sum of squared errors (NSSE) defined in Eq. (2.9). Each ABC–SMC run uses $P = 1000$ particles per generation, a multivariate normal transition kernel with covariance adapted from the previous population, and a quantile tolerance schedule with $\alpha = 0.3$ for up to 10 generations: the tolerance at generation $t+1$ is set to the α-quantile of the accepted distances at generation t.

Quantile tolerance schedule. At each generation t the tolerance ε_t determines the maximum allowable discrepancy for accepting a particle. Rather than fixing ε_t in advance, we adapt it using a quantile schedule: ε_{t+1} is set to the 30th percentile of the distances of the accepted particles at generation t. This adaptive schedule gradually tightens the tolerance, focusing sampling on regions of higher posterior density while maintaining reasonable acceptance rates and avoiding premature loss of particle diversity.

Fairness of calibration across models and locations. The same ABC–SMC configuration (particle count, tolerance schedule, transition kernel, stopping criteria) is used for the baseline model and all behavioral variants across all locations to ensure a fair comparison. No model or location received preferential tolerances, evaluation budgets, or tuning. Consequently, differences in fit quality, inferred parameters, and model probabilities reflect model structure and data characteristics rather than differences in calibration effort.

A4 Synthetic Data Experiment Details

The main text describes the design of the synthetic data experiments: mortality trajectories are generated from behavioral models with varying behavioral sensitivity ζ, small Gaussian noise is added, and both baseline and behavioral models are fitted to these synthetic datasets to assess bias and identifiability. Here we provide implementation details not stated in the main text.

Data-generating parameters. Synthetic mortality trajectories were generated using the behavioral (Mixed) model (Eq. (2.4)) with parameters calibrated to Massachusetts COVID-19 mortality data. The total population size N was fixed to the Massachusetts population, and the parameters π_0, $\mathcal{R}_0$, and δ were set to the weighted medians of their posterior marginal distributions under the behavioral (Mixed) model for Massachusetts. The behavioral sensitivity parameter ζ was varied across six values to represent increasing feedback strength:

$$\zeta \in \{0.001, 0.005, 0.009, 0.012, 0.016, 0.02\}.$$

Each synthetic dataset (for a given ζ and behavioral formulation) was treated as an observed time series and analyzed using the same ABC–SMC configuration as in the real-data analysis (see Section 2.3).

The main text focuses on the behavioral (Mixed) formulation (Fig 4). Supplementary Figs A2 and A3 show the corresponding results when the data-generating model uses the exponential and rational formulations, respectively.

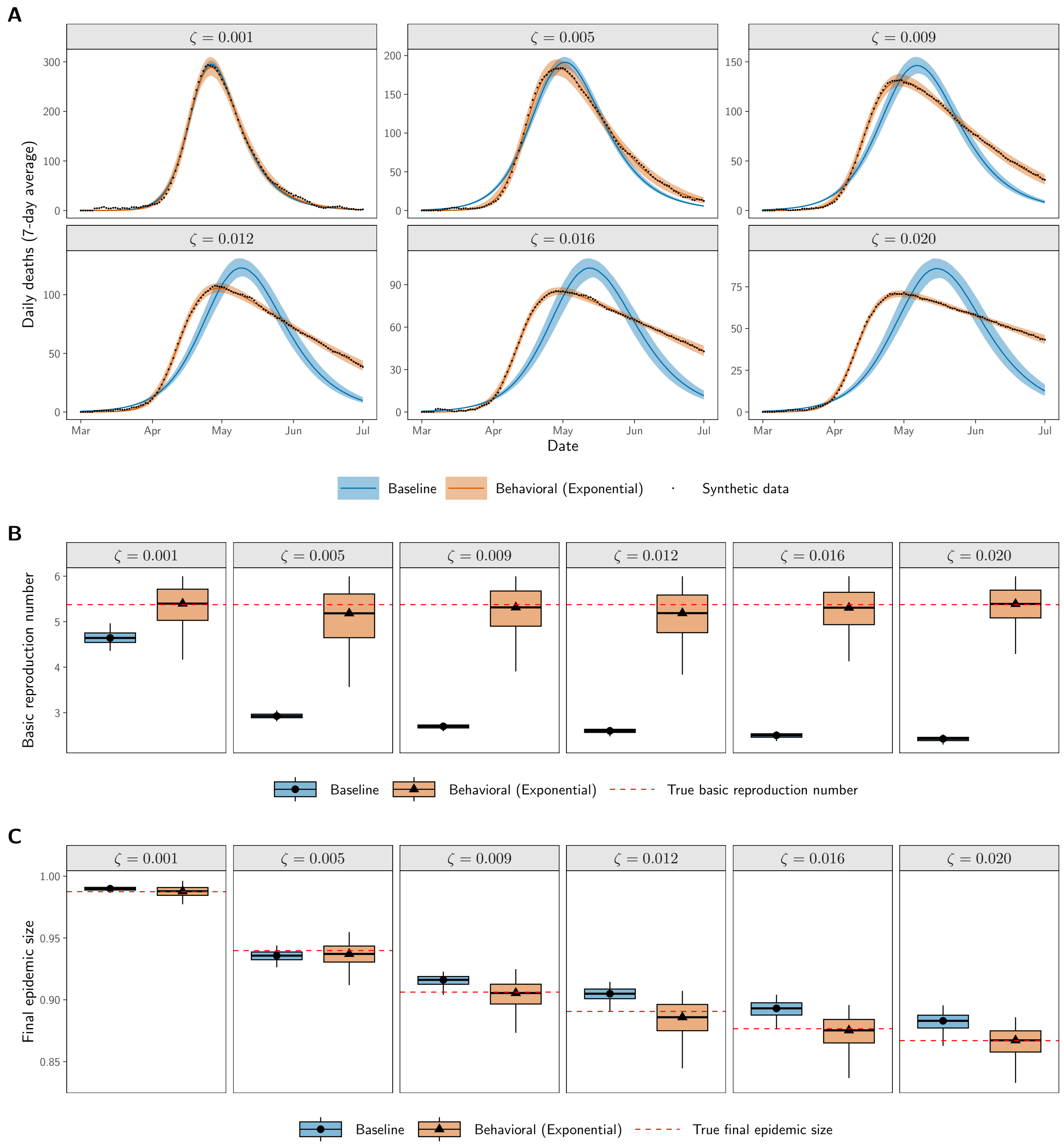

Fig A2. Synthetic data experiment comparing baseline and behavioral (Exponential) models. Synthetic data experiments where mortality trajectories were generated using the behavioral (Exponential) model with known parameters and varying behavioral sensitivity ζ, then fitted with both baseline and behavioral (Exponential) models. **(A)** Posterior predictive fits to synthetic mortality data (black points). Solid lines show posterior medians and shaded regions show 90% credible intervals. The behavioral model accurately recovers the true dynamics across all ζ values, while the baseline model increasingly fails to capture the extended post-peak decline as behavioral feedback strengthens. **(B)** Posterior distributions of the basic reproduction number $\mathcal{R}_0$. The behavioral model recovers the true $\mathcal{R}_0$ (red dashed line) across all scenarios, while the baseline model systematically underestimates $\mathcal{R}_0$, with bias increasing as ζ increases. **(C)** Posterior distributions of the final epidemic size.

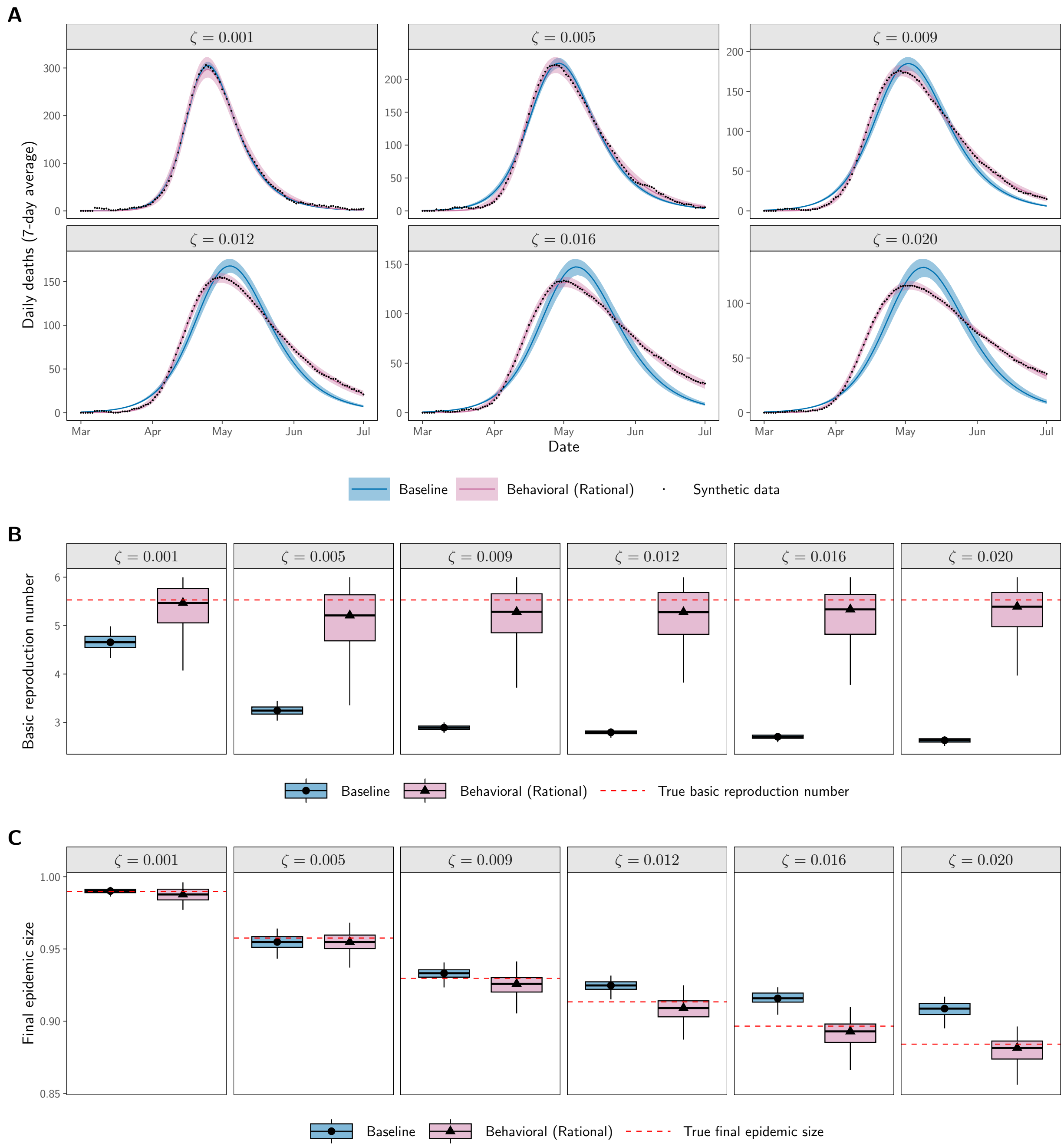

Fig A3. Synthetic data experiment comparing baseline and behavioral (Rational) models. Synthetic data experiments where mortality trajectories were generated using the behavioral (Rational) model with known parameters and varying behavioral sensitivity ζ, then fitted with both baseline and behavioral (Rational) models. **(A)** Posterior predictive fits to synthetic mortality data (black points). Solid lines show posterior medians and shaded regions show 90% credible intervals. The behavioral model accurately recovers the true dynamics across all ζ values, while the baseline model increasingly fails to capture the extended post-peak decline as behavioral feedback strengthens. **(B)** Posterior distributions of the basic reproduction number $\mathcal{R}_0$. The behavioral model recovers the true $\mathcal{R}_0$ (red dashed line) across all scenarios, while the baseline model systematically underestimates $\mathcal{R}_0$, with bias increasing as ζ increases. **(C)** Posterior distributions of the final epidemic size.

A5 Exponential and Rational Behavioral Models

The main text focuses on the behavioral (Mixed) model when presenting posterior predictive fits and comparisons of $\mathcal{R}_0$ and final epidemic size (Figs 2 and 3). Here we provide corresponding summaries for the exponential and rational behavioral formulations.

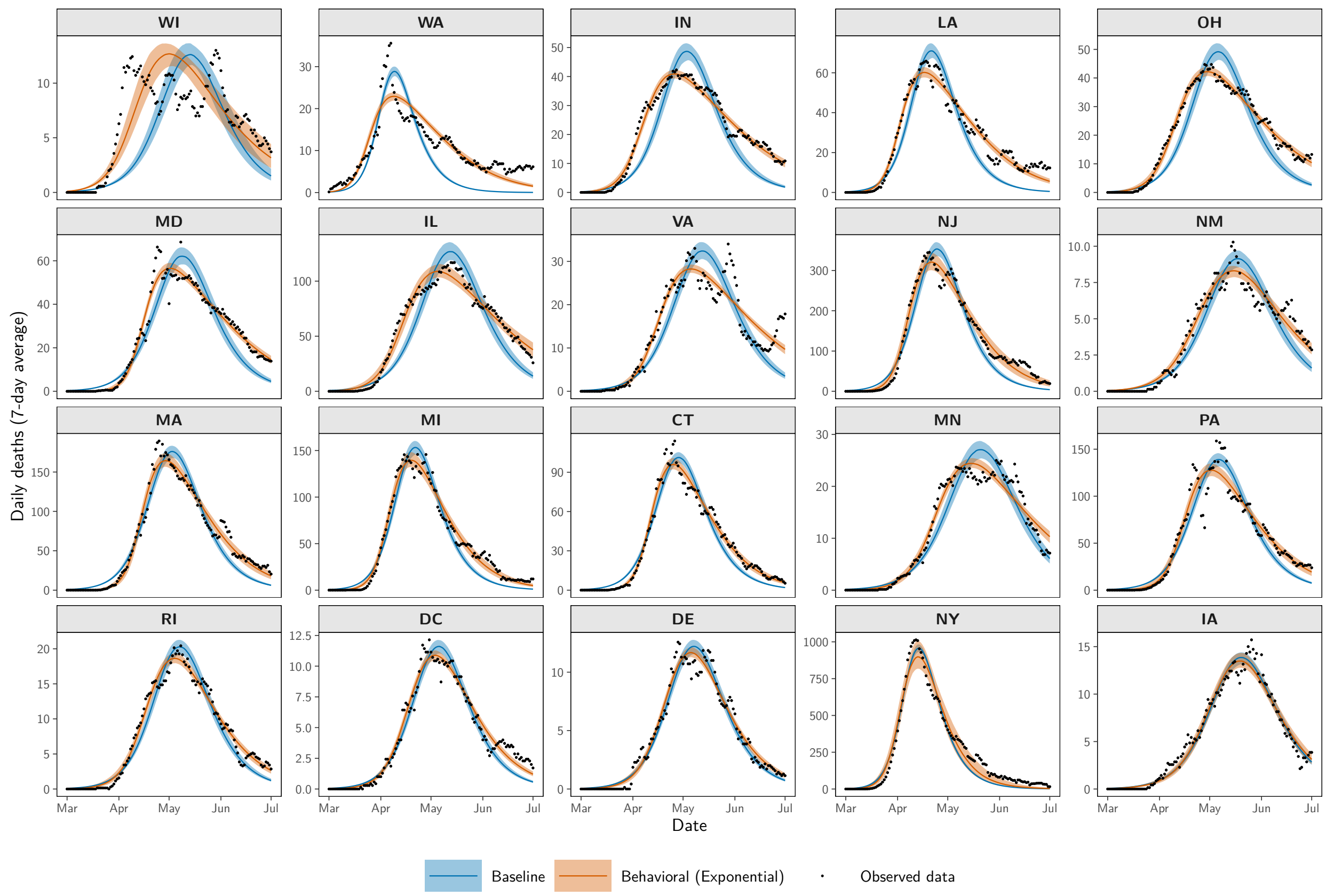

Fig A4. Behavioral (Exponential) model fits compared to baseline across 20 US locations. Posterior predictive fits comparing baseline and behavioral (Exponential) models to 7-day averaged daily COVID-19 deaths during the first pandemic wave (March–July 2020). Black points show observed mortality data. Solid lines show posterior predictive medians and shaded regions show 90% credible intervals (5th–95th percentiles), computed from 1,000 weighted posterior samples from the final ABC–SMC generation. The behavioral (Exponential) model captures the characteristic asymmetric mortality curves with sharp initial rises and gradual extended declines observed in many locations, whereas the baseline model with constant transmission often decays too rapidly. Locations are ordered from left to right and top to bottom by decreasing improvement in fit, measured by the difference in median posterior NSSE between the behavioral and baseline models, analogous to Fig 2 in the main text.

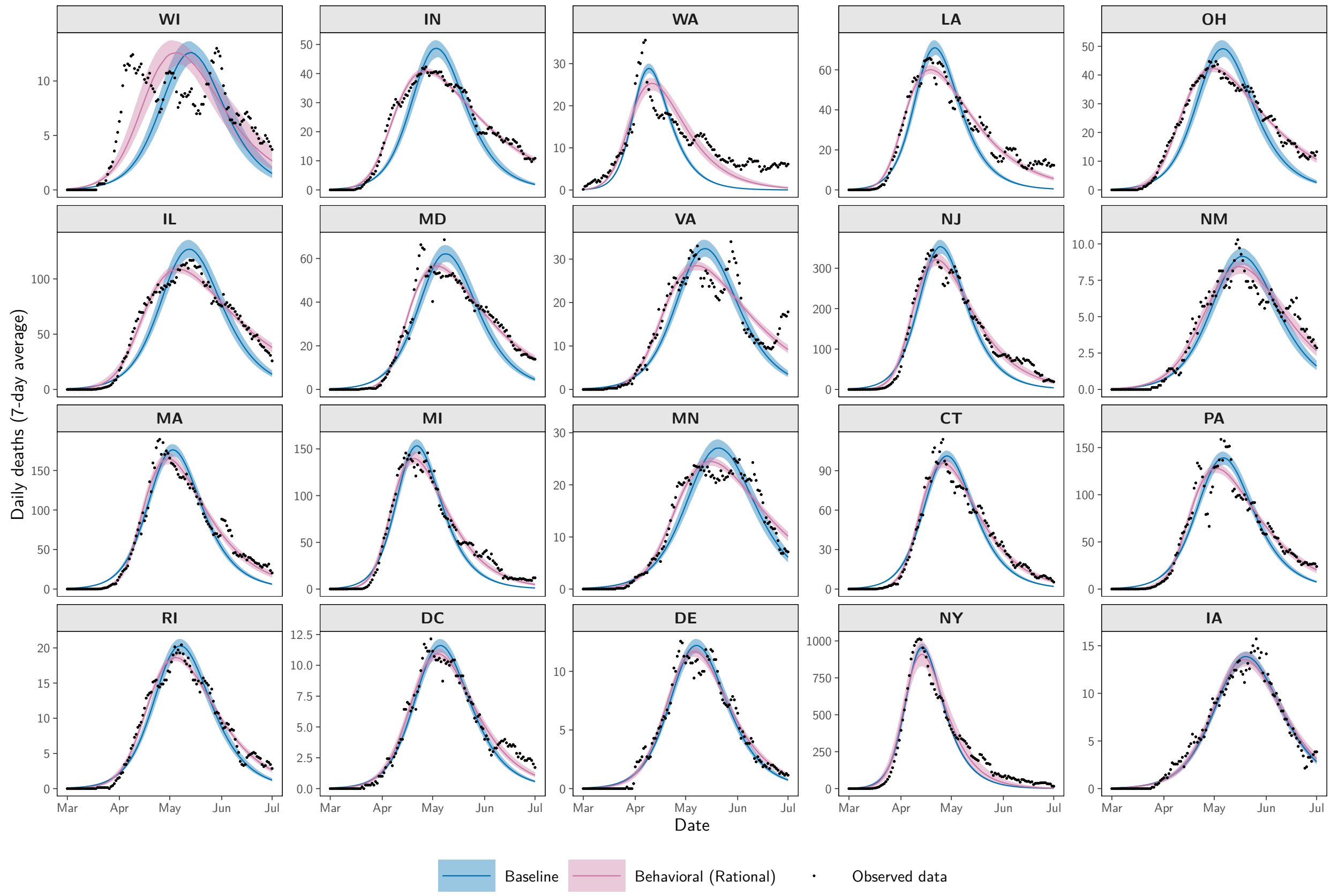

Fig A5. Behavioral (Rational) model fits compared to baseline across 20 US locations. Posterior predictive fits comparing baseline and behavioral (Rational) models to 7-day averaged daily COVID-19 deaths during the first pandemic wave (March–July 2020). Black points show observed mortality data. Solid lines show posterior predictive medians and shaded regions show 90% credible intervals (5th–95th percentiles), computed from 1,000 weighted posterior samples from the final ABC–SMC generation. The behavioral (Rational) model also reproduces the asymmetric mortality curves with sharp rises and extended declines that are difficult to match under a constant-transmission baseline model. Locations are ordered from left to right and top to bottom by decreasing improvement in fit, measured by the difference in median posterior NSSE between the behavioral and baseline models, analogous to Fig 2 in the main text.

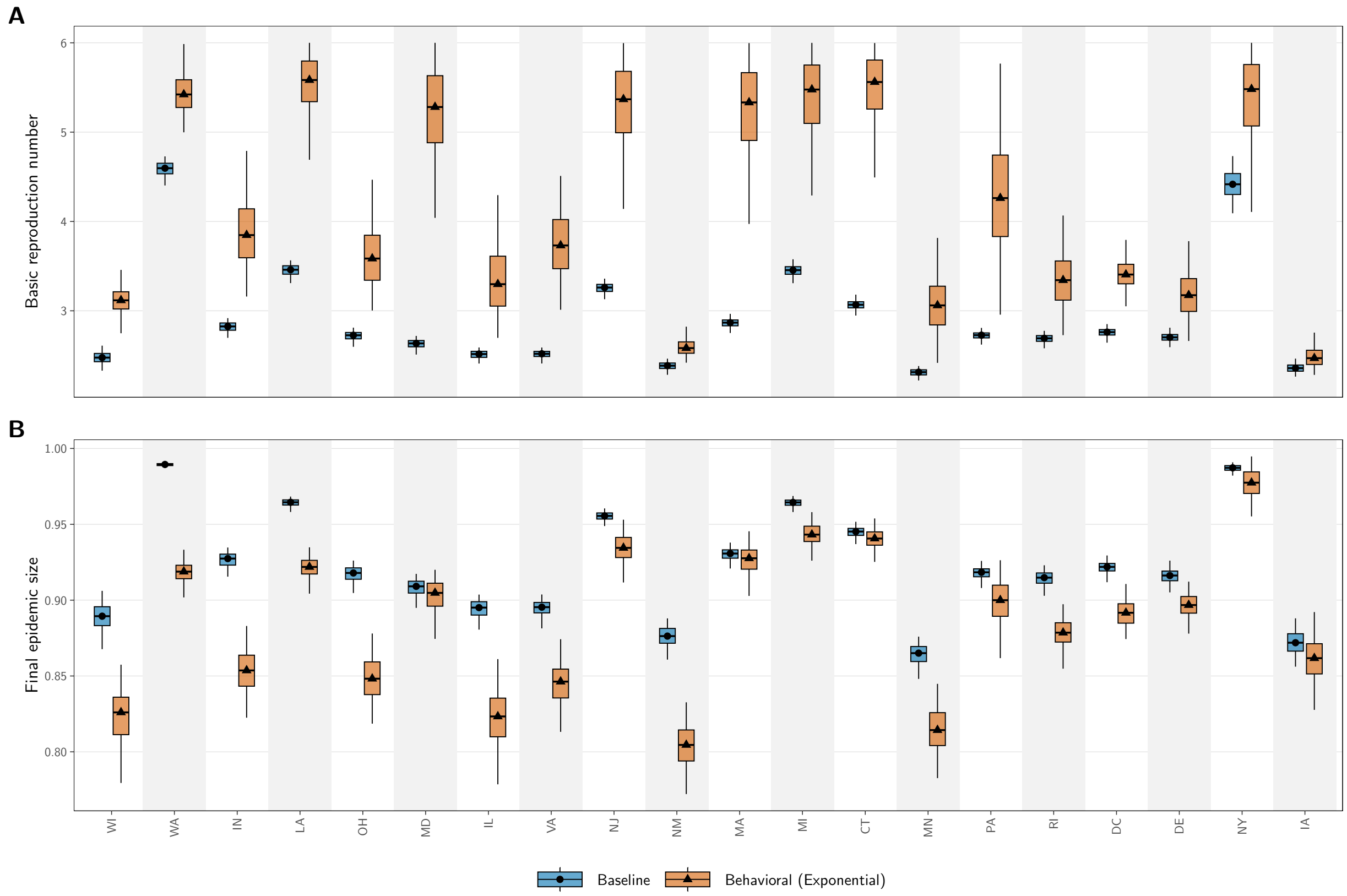

Fig A6. Baseline versus behavioral (Exponential) model estimates of $\mathcal{R}_0$ and final epidemic size. Posterior distributions of the basic reproduction number $\mathcal{R}_0$ and final epidemic size for the baseline and behavioral (Exponential) models fitted to COVID-19 mortality data from 20 US locations during the first pandemic wave (March–July 2020). Boxplots show median, interquartile range, and whiskers extending to $1.5 \times$ IQR within the support of weighted posterior samples. Locations are ordered from left to right by decreasing difference in median NSSE between the baseline and behavioral (Exponential) models.

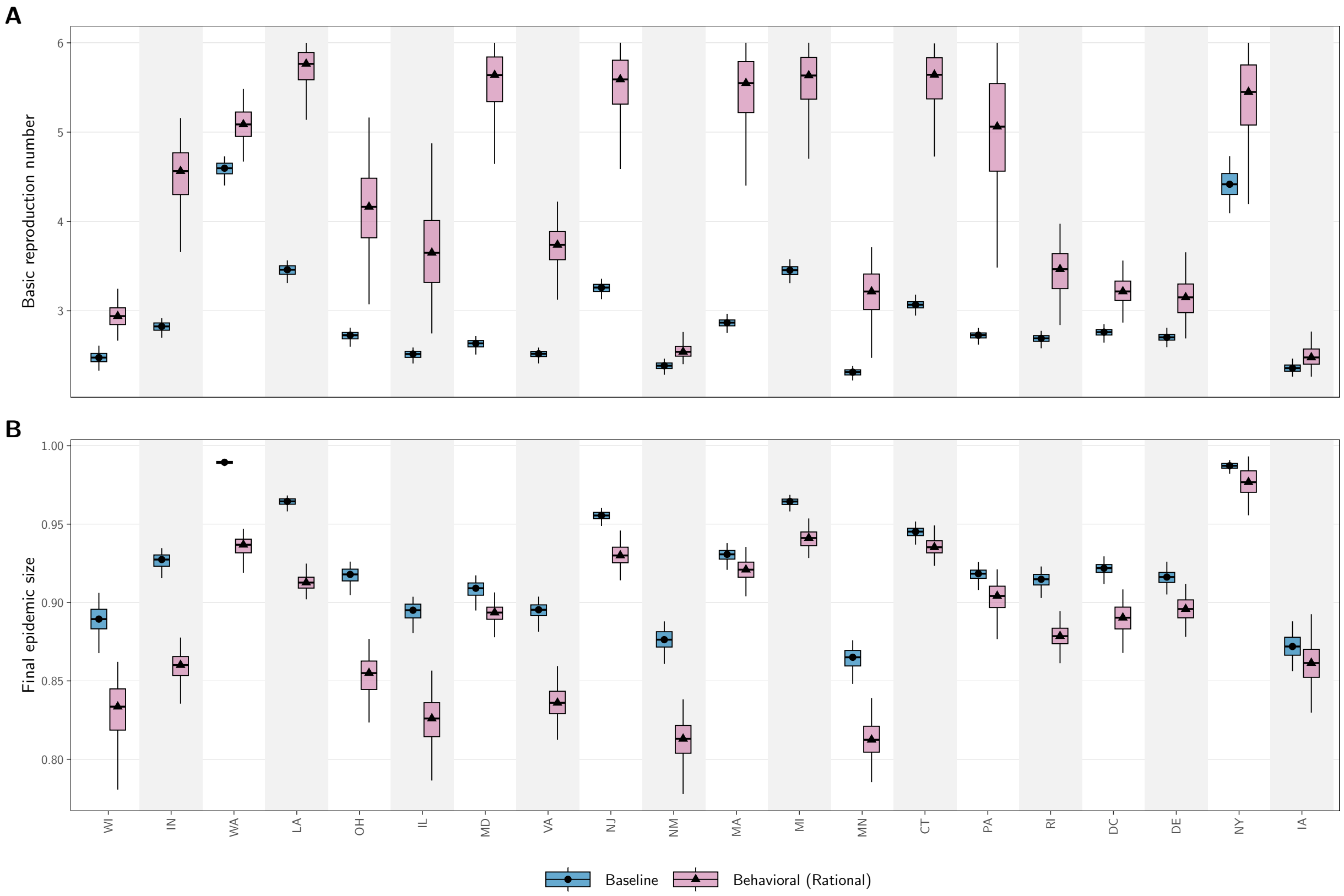

Fig A7. Baseline versus behavioral (Rational) model estimates of $\mathcal{R}_0$ and final epidemic size. Posterior distributions of the basic reproduction number $\mathcal{R}_0$ and final epidemic size for the baseline and behavioral (Rational) models fitted to COVID-19 mortality data from 20 US locations during the first pandemic wave (March–July 2020). Boxplots show median, interquartile range, and whiskers extending to 1.5 × IQR within the support of weighted posterior samples. Locations are ordered from left to right by decreasing difference in median NSSE between the baseline and behavioral (Rational) models.

Table A6. Comparison of the baseline model and the behavioral (Exponential) model across 20 US locations. For each location, Median $\mathrm{NSSE_B}$ and Median $\mathrm{NSSE_E}$ denote the median posterior normalized sum of squared errors (NSSE) under the baseline B and behavioral (Exponential) E models respectively. $\mathrm{BF_{eq}}$ is the Bayes factor under equal model priors in favor of the exponential model versus the baseline model at the stabilized ABC–SMC generation, and "Evidence" gives the corresponding verbal strength of evidence. Table is ordered by decreasing Bayes factor in favor of the behavioral model.

Location	Median $\mathrm{NSSE_B}$	Median $\mathrm{NSSE_E}$	$\mathrm{BF_{eq}}$	Evidence
VA	0.0815	**0.0385**	9343.72	very strong
RI	0.0251	**0.0107**	529.25	very strong
WI	0.2540	**0.1144**	335.87	very strong
OH	0.0685	**0.0053**	190.33	very strong
NM	0.0493	**0.0189**	124.12	strong
IN	0.0900	**0.0086**	104.37	strong
CT	0.0331	**0.0106**	69.45	strong
IL	0.0574	**0.0111**	61.22	strong
MN	0.0398	**0.0184**	59.66	strong
MD	0.0617	**0.0146**	26.79	strong
PA	0.0472	**0.0311**	20.64	strong
NJ	0.0430	**0.0125**	18.37	positive
MA	0.0377	**0.0133**	16.73	positive
DE	0.0214	**0.0164**	16.53	positive
LA	0.0837	**0.0160**	16.33	positive
WA	0.1784	**0.0675**	8.05	positive
MI	0.0345	**0.0112**	8.05	positive
DC	0.0286	**0.0144**	1.17	very weak
IA	**0.0139**	0.0161	0.79	favors Baseline
NY	0.0277	**0.0263**	0.03	favors Baseline

Table A7. Comparison of the baseline model and the behavioral (Rational) model across 20 US locations. For each location, Median NSSE_B and Median NSSE_R denote the median posterior normalized sum of squared errors (NSSE) under the baseline B and behavioral (Rational) R models respectively. BF_eq is the Bayes factor under equal model priors in favor of the rational model versus the baseline model at the stabilized ABC–SMC generation, and "Evidence" gives the corresponding verbal strength of evidence. Table is ordered by decreasing Bayes factor in favor of the behavioral model.

Location	Median NSSE_B	Median NSSE_R	BF_eq	Evidence
MN	0.0398	**0.0178**	766.21	very strong
MI	0.0345	**0.0112**	618.29	very strong
RI	0.0251	**0.0098**	580.79	very strong
VA	0.0815	**0.0384**	551.33	very strong
LA	0.0837	**0.0168**	484.47	very strong
MD	0.0617	**0.0153**	260.53	very strong
WI	0.2540	**0.1431**	235.46	very strong
IL	0.0574	**0.0087**	147.28	strong
IN	0.0900	**0.0070**	99.39	strong
OH	0.0685	**0.0045**	75.25	strong
PA	0.0472	**0.0292**	49.68	strong
MA	0.0377	**0.0138**	23.95	strong
NJ	0.0430	**0.0124**	22.78	strong
NM	0.0493	**0.0219**	21.02	strong
WA	0.1784	**0.1001**	20.84	strong
DE	0.0214	**0.0162**	15.75	positive
IA	**0.0139**	0.0159	2.37	very weak
CT	0.0331	**0.0122**	1.76	very weak
DC	0.0286	**0.0159**	1.15	very weak
NY	0.0277	**0.0265**	0.04	favors Baseline

A6 Effective Transmission Rate

For each model variant and location, we computed the posterior distribution of the effective transmission rate $\beta(t)$ by propagating uncertainty from the ABC–SMC parameter posteriors. From each location- and model-specific posterior, we drew 1,000 parameter sets, where the probability of selecting a posterior particle is proportional to its ABC–SMC importance weight. For each sampled parameter set we simulated the SEIRD model, extracted the aligned infectious trajectory $I(t)$, and computed the corresponding effective transmission rate

$$\beta_{\text{eff}}(t) = \begin{cases} \beta_0, & \text{Baseline,} \\ \beta_0\, e^{-\zeta\,\delta\, I(t)}, & \text{Behavioral (Exponential),} \\ \frac{\beta_0}{1+\zeta\,\delta\, I(t)}, & \text{Behavioral (Rational),} \\ \frac{\beta_0\, e^{-\zeta\,\delta\, I(t)}}{1+\zeta\,\delta\, I(t)}, & \text{Behavioral (Mixed).} \end{cases}$$

Pointwise 5th, 50th, and 95th percentiles across the ensemble of 1,000 trajectories form the median curve and the 90% pointwise credible interval bands shown in Figs A8, A9, and A10.

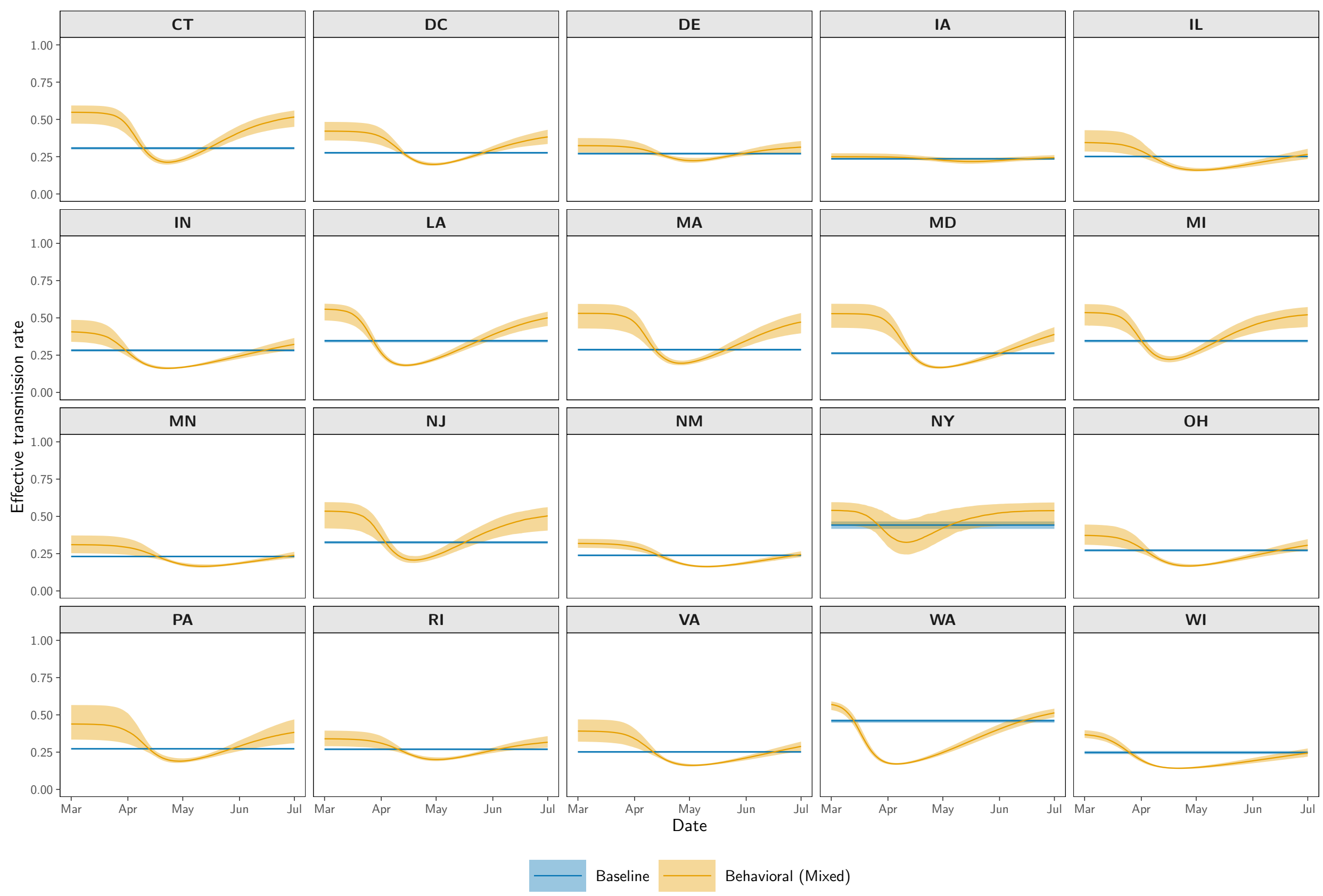

Fig A8. Effective transmission rate $\beta(t)$ under the Behavioral (Mixed) model. Shown are posterior median trajectories together with 90% pointwise credible intervals, obtained by propagating weighted ABC–SMC posterior samples through the SEIRD model to compute $\beta(t)$ for each draw.

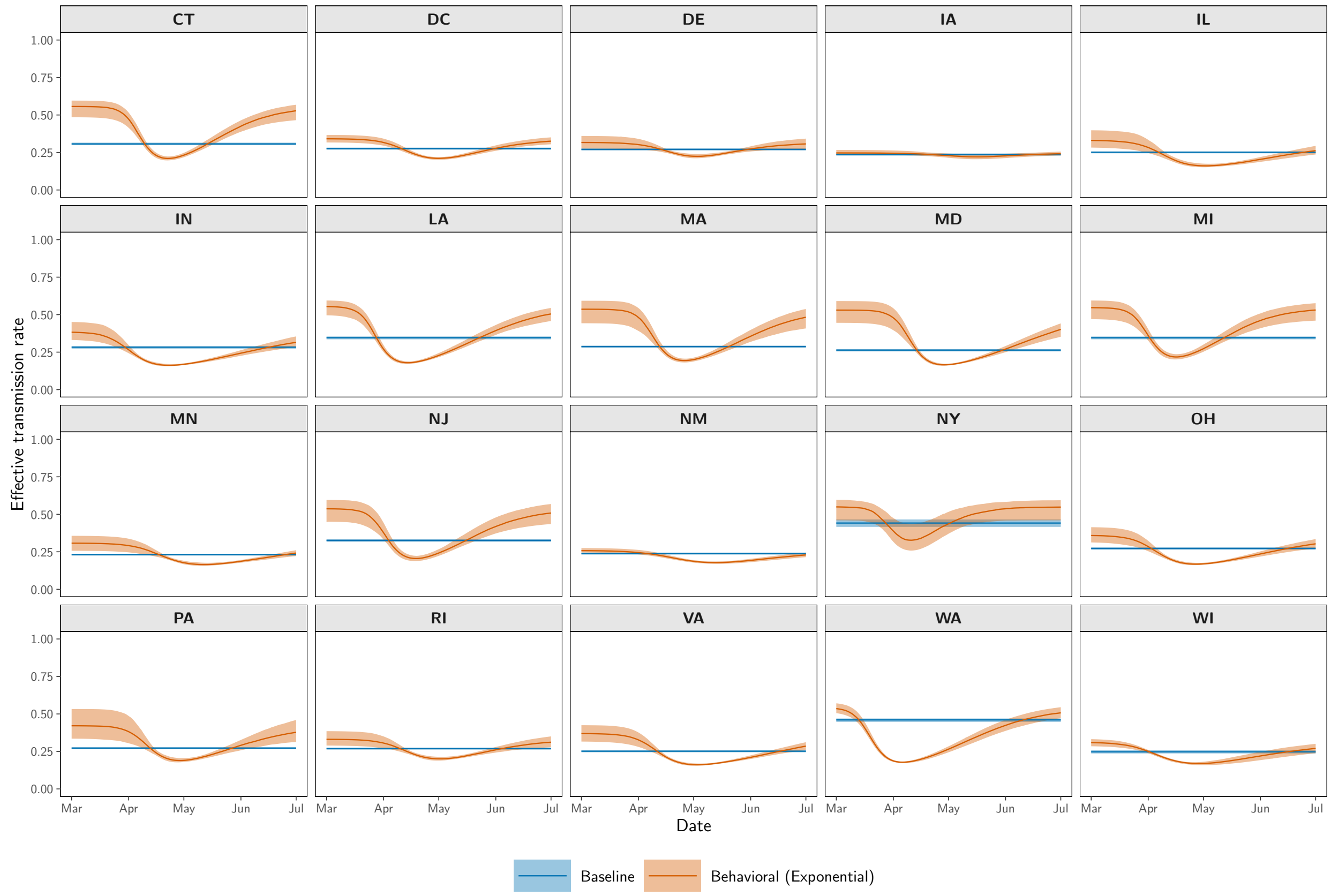

Fig A9. Effective transmission rate $\beta(t)$ under the Behavioral (Exponential) model. Shown are posterior median trajectories together with 90% pointwise credible intervals, obtained by propagating weighted ABC–SMC posterior samples through the SEIRD model to compute $\beta(t)$ for each draw.

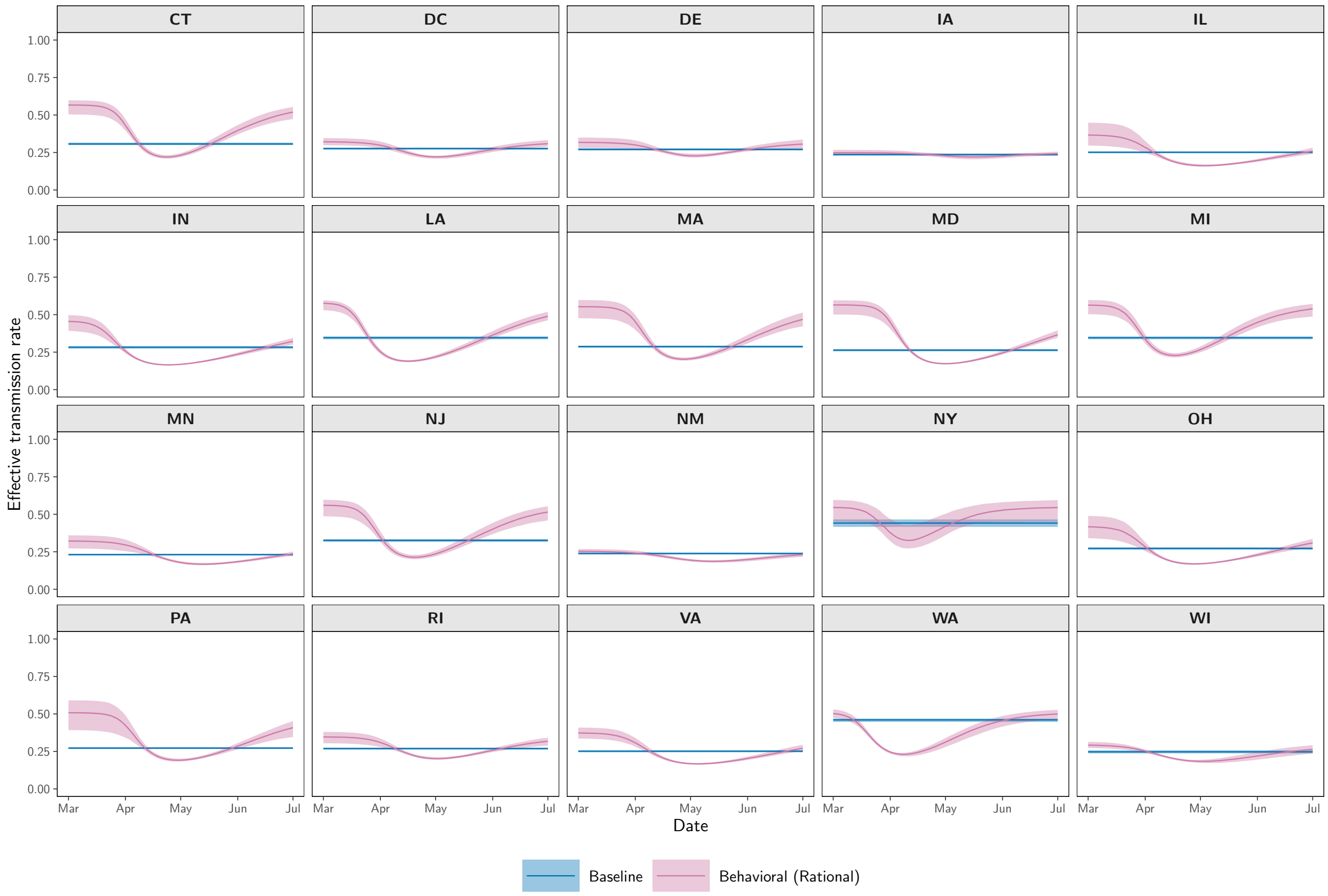

Fig A10. Effective transmission rate $\beta(t)$ under the Behavioral (Rational) model. Shown are posterior median trajectories together with 90% pointwise credible intervals, obtained by propagating weighted ABC–SMC posterior samples through the SEIRD model to compute $\beta(t)$ for each draw.

A7 Effective Reproduction Number

For each model and location, we compute the effective reproduction number $\mathcal{R}_e(t)$ as

$$\mathcal{R}_e(t) = \mathcal{R}_0(t)\,\frac{S(t)}{N},$$

where $S(t)$ is the susceptible population and N is the total population, as defined in Eq. **2.7**.

To quantify uncertainty, we propagate posterior parameter uncertainty by drawing 1,000 weighted samples from each model's ABC–SMC posterior. For each sampled parameter set, we simulate the SEIRD model, extract the aligned trajectories $S(t)$ and $I(t)$, compute $\mathcal{R}_e(t)$, and summarize across the ensemble using the 5th, 50th, and 95th percentiles. These form the posterior median curves and 90% pointwise credible intervals shown in Figs A11, A12, and A13.

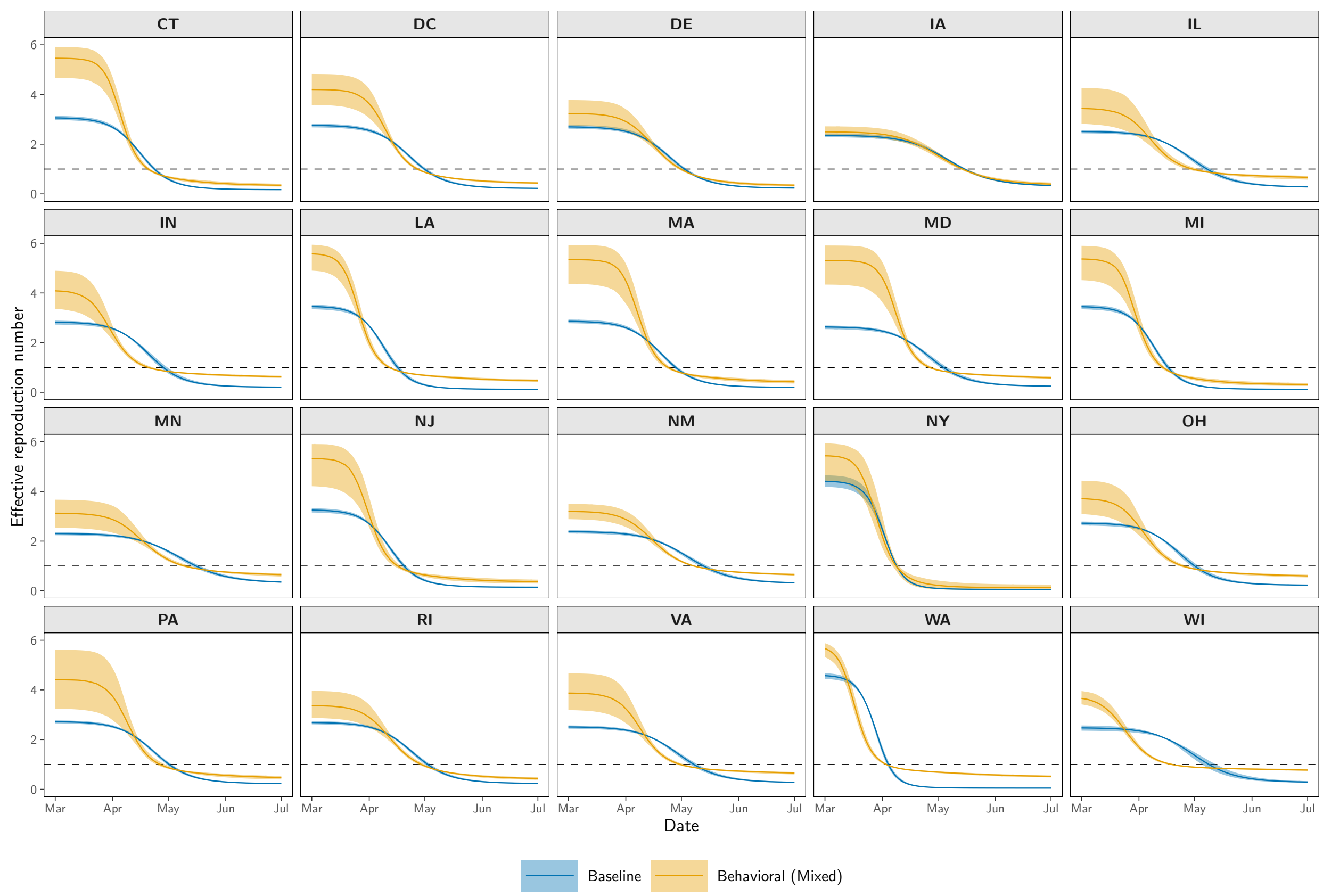

Fig A11. Posterior effective reproduction number $\mathcal{R}_e(t)$ under the Behavioral (Mixed) model. Posterior median curves and 90% pointwise credible intervals are shown for all locations.

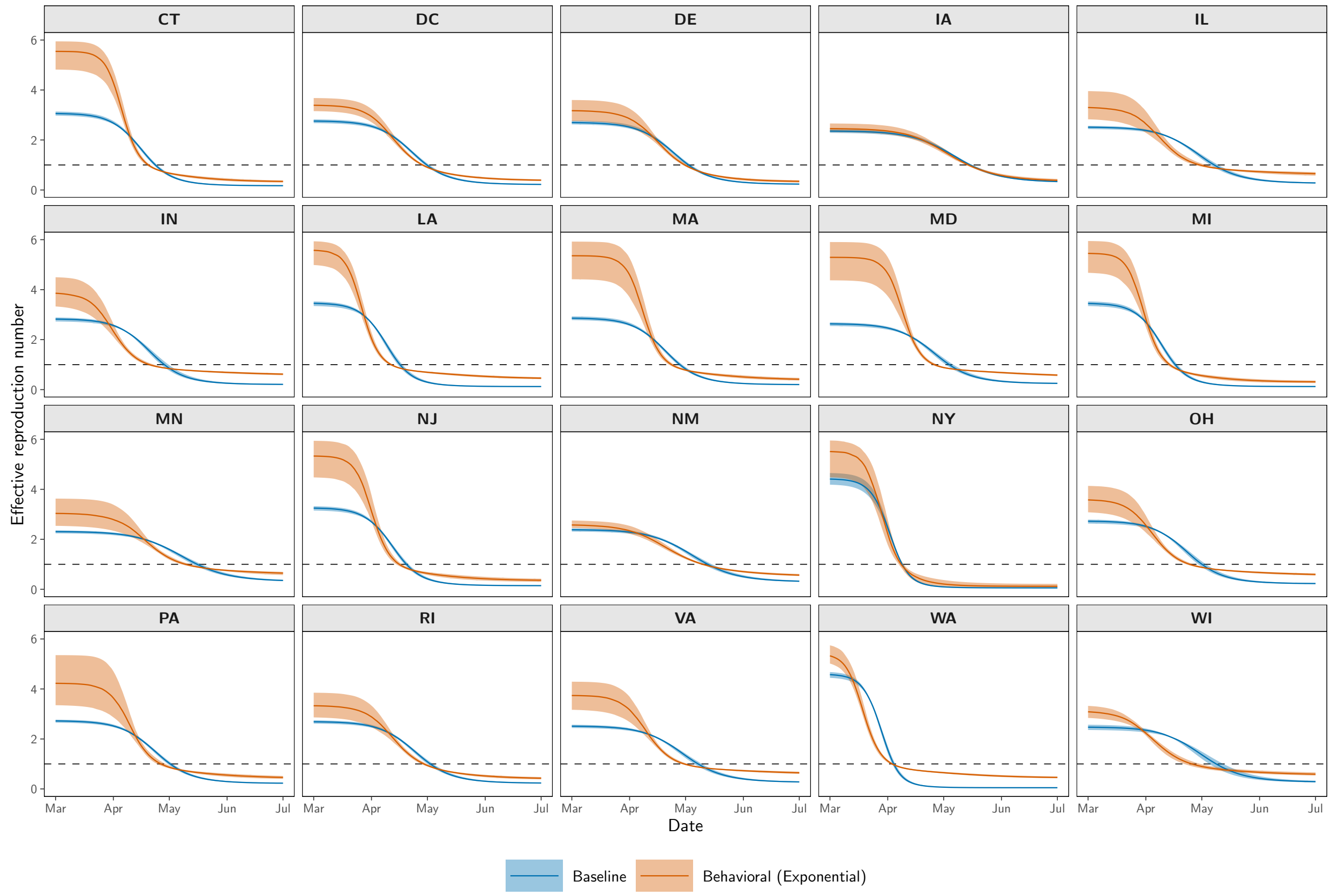

Fig A12. Posterior effective reproduction number $\mathcal{R}_e(t)$ under the Behavioral (Exponential) model. Posterior median curves and 90% pointwise credible intervals are shown for all locations.

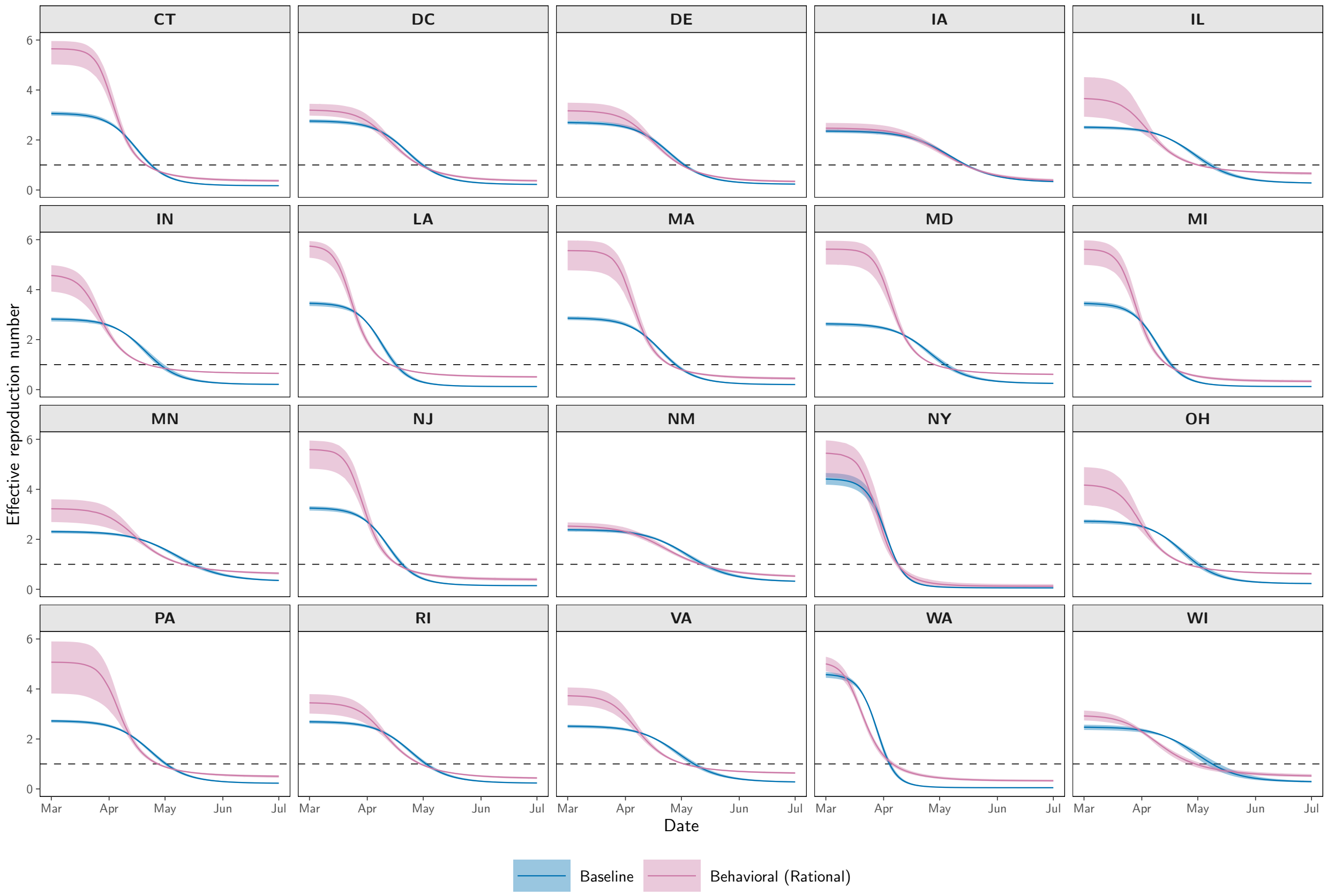

Fig A13. Posterior effective reproduction number $\mathcal{R}_e(t)$ under the Behavioral (Rational) model. Posterior median curves and 90% pointwise credible intervals are shown for all locations.

References

[1] Lauer SA, Grantz KH, Bi Q, Jones FK, Zheng Q, Meredith HR, et al. The incubation period of coronavirus disease 2019 (COVID-19) from publicly reported confirmed cases: estimation and application. Annals of internal medicine. 2020;172(9):577-82. doi:10.7326/M20-0504.

[2] He X, Lau EH, Wu P, Deng X, Wang J, Hao X, et al. Temporal dynamics in viral shedding and transmissibility of COVID-19. Nature Medicine. 2020;26(5):672-5. doi:10.1038/s41591-020-0869-5.

[3] Wölfel R, Corman VM, Guggemos W, Seilmaier M, Zange S, Müller MA, et al. Virological assessment of hospitalized patients with COVID-2019. Nature. 2020;581(7809):465-9. doi:10.1038/s41586-020-2196-x.

[4] Puhach O, Meyer B, Eckerle I. SARS-CoV-2 viral load and shedding kinetics. Nature Reviews Microbiology. 2023;21(3):147-61. doi:10.1038/s41579-022-00822-w.
